# A Review on Nano Ti-Based Oxides for Dark and Photocatalysis: From Photoinduced Processes to Bioimplant Applications

**DOI:** 10.3390/nano13060982

**Published:** 2023-03-08

**Authors:** Christine Joy Querebillo

**Affiliations:** Leibniz-Institute for Solid State and Materials Research (IFW) Dresden, Helmholtzstr. 20, 01069 Dresden, Germany; c.j.querebillo@ifw-dresden.de

**Keywords:** reactive oxygen species, photocatalytic efficiency, charge separation, light harvesting, black TiO_2_, titanium alloys, residual disinfection, dark catalysis, antibacterial, inflammation

## Abstract

Catalysis on TiO_2_ nanomaterials in the presence of H_2_O and oxygen plays a crucial role in the advancement of many different fields, such as clean energy technologies, catalysis, disinfection, and bioimplants. Photocatalysis on TiO_2_ nanomaterials is well-established and has advanced in the last decades in terms of the understanding of its underlying principles and improvement of its efficiency. Meanwhile, the increasing complexity of modern scientific challenges in disinfection and bioimplants requires a profound mechanistic understanding of both residual and dark catalysis. Here, an overview of the progress made in TiO_2_ catalysis is given both in the presence and absence of light. It begins with the mechanisms involving reactive oxygen species (ROS) in TiO_2_ photocatalysis. This is followed by improvements in their photocatalytic efficiency due to their nanomorphology and states by enhancing charge separation and increasing light harvesting. A subsection on black TiO_2_ nanomaterials and their interesting properties and physics is also included. Progress in residual catalysis and dark catalysis on TiO_2_ are then presented. Safety, microbicidal effect, and studies on Ti-oxides for bioimplants are also presented. Finally, conclusions and future perspectives in light of disinfection and bioimplant application are given.

## 1. Introduction

Titanium dioxide (TiO_2_), or titania, occurs naturally and forms spontaneously when bare Ti is exposed to air [1,2]. At room temperature, TiO_2_ is commonly considered an *n*-type semiconductor [3,4] with a bandgap (*E_g_*) of around 3 eV (3.2 eV for anatase, 3 eV for rutile) [2,4]. Since the discovery of its photoelectrochemical (PEC) water-splitting ability by Fujishima and Honda [3,5], TiO_2_ remains today as the standard photocatalyst. The excitation of its electrons from the valence band (VB) to the conduction band (CB) upon exposure to light with energy *E* ≥ *E_g_*, i.e., UV (to violet, 413 nm) light for the bulk form, results in photogenerated charge carriers (electrons and holes) which can be utilized in various processes, resulting in its photocatalytic activity. In addition, TiO_2_ exhibits desirable material properties, such as its wide availability, biocompatibility, chemical stability in an aqueous environment, and affordability [6,7,8,9,10,11,12,13,14], resulting in the application of TiO_2_ in different fields, such as catalysis, alternative/clean energy technologies, environmental cleaning, pollutant degradation, medicine, pharmaceuticals, disinfection, and biomedical implants [1,2,3,4,6,7,8,9,10,11,12,13,14,15,16,17,18,19,20,21].

**Figure 1:**
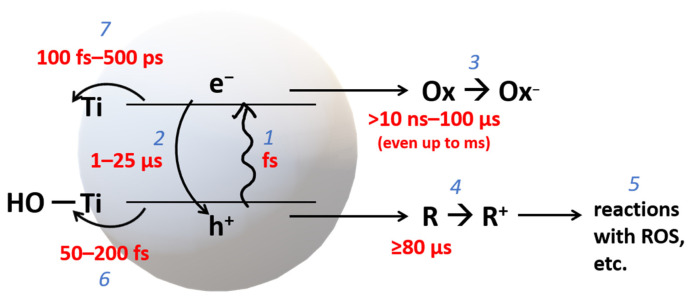
The core steps in photocatalytic mechanism include (1) absorption of light which forms charge carriers, (2) recombination of charge carriers, (3) reductive process with electrons in the conduction band (CB), (4) oxidative pathway undertaken by a valence-band (VB) hole, (5) further reactions (hydrolysis, reactions with reactive oxygen species (ROS), etc.), (6) trapping a VB hole at a Ti-OH group on the surface, and (7) trapping of CB electron at a Ti(IV) site to produce Ti(III). Redrawn from ref. [26] to include timescales obtained from [22,23,24,25,26]. The timescale in 3 and 4 are mainly from nontrapped electrons and holes (i.e., directly from the CB and VB).

The mechanism of photocatalysis on TiO_2_ depends on the type of catalytic reaction that is being investigated, though it primarily involves interfacial (and bulk) processes of the photoinduced electrons and holes, which occur at different time scales (Figure 1) [22,23,24,25,26]. The faster charge carrier generation due to photon absorption (1) compared to electron–hole recombination (2) enables the possibility of having CB electrons and VB holes that can be tapped for reductive (3) and oxidative (4) processes, respectively. However, the recombination process lies at the same time scale as these interfacial charge transfer (CT) processes, and therefore efforts to further delay the recombination process can improve the photocatalytic efficiency of TiO_2_.

Furthermore, the VB holes can transport quickly to hole trap sites at the surface, such as at surface Ti-OH groups (6), which is often the case for the photogenerated holes because of the fast femtosecond process. Consequently, free VB holes are scarcely present in TiO_2_ [24], and surface-trapped holes are usually responsible for the oxidation reaction which can occur faster in the ps–ns range [22,23,24,25,26]. The electrons, on the other hand, can get trapped at Ti(IV) sites (7) and form Ti(III), which can also participate in other redox processes [22,23,24,25,26].

**Figure 5:**
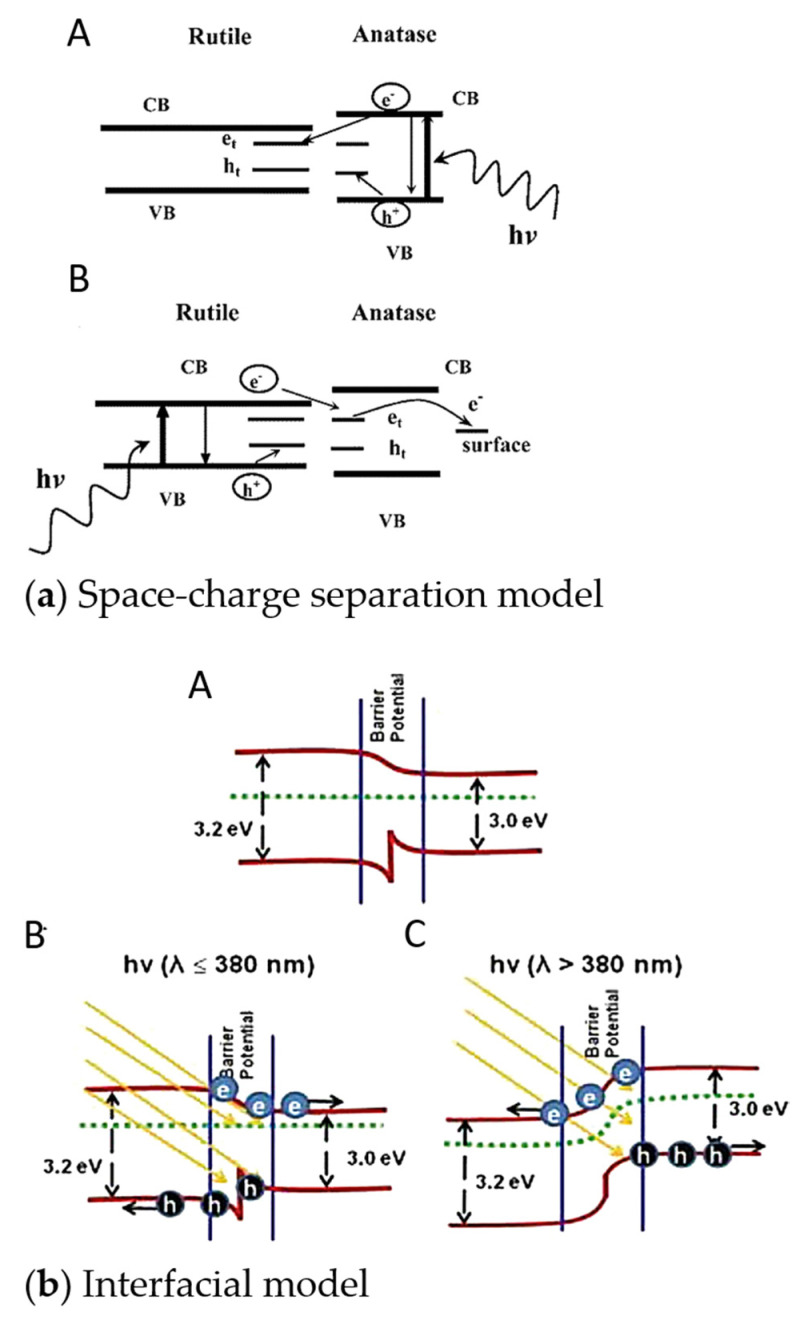
Models describing the mixed phase synergistic effect due to heterojunction formation: (**a**) space-charge separation model in which the transfer of electron can occur from (A) anatase to rutile or (B) rutile to anatase. Reprinted (adapted) with permission from Hurum, D.C., et al. Copyright 2003 American Chemical Society [163]; (**b**) Interfacial model which describes the band bending at the interface of anatase and rutile (A) at equilibrium, (B) when irradiated with light of wavelength ≤380 nm, and (C) when irradiated with >380 nm. Adapted and reprinted with permission from ref. [164]. Copyright 2011 Elsevier B.V.

Direct recombination of the photogenerated carriers usually does not occur and instead happens upon meeting trapped complementary charge carriers. For example, mobile electrons can recombine with trapped holes, decreasing the former’s lifetime and reducing the photocatalytic performance of TiO_2_. As such, anatase usually performs better as a photocatalyst than rutile due to its longer electron lifetime (>few ms for anatase vs. ~24 ns for rutile) and stronger band bending [27,28]. Further photocatalytic reactions on TiO_2_ occur on the surface involving reactive oxygen species (ROS) (Figure 1, (5)) [22].

In the past decades, most studies on TiO_2_ are focused on its photocatalytic activity (both in the bulk and nanomaterial form), and the literature is overflowing with strategies to improve its performance by addressing inherent deterrents, extending the spectral range for which TiO_2_-based photocatalysis can be used, or enhancing the light utilization through various geometries (nanotextures, structured arrays, etc.). These improvements are based mainly on the key steps in photocatalysis, focusing on light absorption, generation of charge carriers, their separation and transport, catalyst replenishment, and prevention of back and side processes. With the goal and steps clarified, TiO_2_ photocatalysis still faces a number of challenges considering that modern materials require multiple functionalities and therefore a balance of its properties. Hence, the influence of different TiO_2_ properties on its photocatalytic performance is also a major focus of research.

Almost overshadowed by the numerous works on photocatalysis but persisting mainly due to the excellent biocompatibility and oxidative bleaching ability of TiO_2_, research on TiO_2_ in the “dark” or in the absence of light has also been growing steadily over the years. Titanium, from which TiO_2_ can be grown, exhibits excellent mechanical properties which helped in establishing its place in the field of biomedical implants. For example, titanium and its alloys are standard materials used for bone implants, and strong research in medicine and engineering is focused on continuously improving their physicochemical properties, mechanical properties, and designability/processability [29,30,31,32,33], on top of added functionalities desired in modern biomaterials, such as its antimicrobial and regenerative properties [34,35,36,37,38,39]. The last two are also attributed to its catalytic ROS-forming ability, which should then be considered in bioimplant applications. After implantation surgery, i.e., in the postoperative phase, implant material surfaces are devoid of light. Understanding the mechanism of “dark” catalysis on TiO_2_ in the physiological condition is therefore important in addressing the current challenges and limitations not only for Ti and TiO_2_ but also for more advanced Ti alloys and Ti-based materials and their oxides for implant application.

Considering the vast use of TiO_2_, it is not surprising that the published works on TiO_2_ catalysis come from many different disciplines of different perspectives. Modern scientific and engineering challenges then often entail a multidisciplinary approach to answer the increasingly becoming more complex questions. Yet, if we look at the literature, it seems that there is still a need to consolidate this immense knowledge of TiO_2_ catalysis from a multidisciplinary perspective.

The different knowledge and experiences we gain by working on different topics involving titania can help us develop the skill of understanding catalysis on TiO_2_ nanomaterials from a more inclusive viewpoint. Different fields working on titania, for instance, electromagnetic field enhancement on semiconductors [40,41,42,43,44,45,46,47,48,49,50], dye photodegradation (see Section 2.2), and bioimplant applications [31,36,51,52,53,54,55,56] to name a few, may all involve catalytic properties of TiO_2_ nanomaterials, yet they also need a nuanced understanding of TiO_2_ in light of specific, targeted applications. Such background and experience can certainly help us easily understand the literature though regularly immersing oneself in various literature on TiO_2_ catalysis easily available to us nowadays, and constantly discussing with colleagues and peers can also help us be familiarized and updated with the progress on TiO_2_ catalysis. As such, despite the experience and background of the author, which certainly helped in the reading and analysis of the literature for this review, the method of regularly keeping up to date with the literature, engaging in scientific discussions, and a period of intensive gathering and reading of the literature on TiO_2_ catalysis was therefore adapted in preparing this review. Many excellent works and reviews helped in the preparation of a general survey, with emphasis on certain points of interest—namely, the advancements in photocatalytic enhancement, the photocatalytic and dark bactericidal activities, and the use of Ti (and Ti-based) oxide nanomaterials for bioimplants. In some topics, the readers are referred to excellent reviews available in the literature, and the scope is limited to oxides of Ti (mainly TiO_2_). On the other hand, the preparation of this review was conducted with the awareness that the answers to present-day complex scientific questions may still not be available in the literature, and due to the multidisciplinary nature of these questions and the explosion of the available literature on the internet, not all available review materials on the internet and in print can be included in this review. Nevertheless, inspired by the present challenges that the bioimplant community wishes to address, a review on photo- and dark catalysis of TiO_2_ is presented here.

In this review, the catalysis of TiO_2_ nanomaterials, both in the presence of light (photocatalysis) and in the dark, is presented to give a general overview of the full spectrum of its catalytic activity. A section discussing the role of ROS in TiO_2_ photocatalysis, which is crucial in photo- and dark catalysis on TiO_2_, is included. As the understanding of the role of ROS in photocatalysis is more extensive, it is beneficial to look at it from a mechanistic perspective without going into details, as excellent reviews and articles also exist in the literature [22,57,58]. The influence of some properties of TiO_2_ on its performance in photocatalysis will then be presented to understand the surface engineering that has been carried out to advance the photocatalysis field. TiO_2_ nanoparticles (NPs), having been extensively developed for photocatalysis, also pose some risks, and the safety of using them will also be discussed. Together with this, the other side of the coin, the photocatalytic antibacterial property afforded by TiO_2_ NPs, is presented. Then, highlights and advancements in the efforts to boost the photocatalytic efficiency of TiO_2_ mainly in terms of charge separation enhancement and improvements in light harvesting will be presented. Black TiO_2_ nanomaterials, a current hot topic in the field of TiO_2_ photocatalysis, will also be presented in light of their physics and photocatalytic activity.

Many studies on dark catalysis are investigations on the influence of Ti-based implants on the inflammatory response and vice versa. In addition, early studies on dark catalysis are observations carried out mainly as a reference to photocatalytic works and the residual effect after the removal of irradiation. These will be presented to serve as a bridge between photo- and “dark catalysis” and will be followed by studies addressing and contributing to the so-far understanding of the dark catalysis mechanism. Dark catalysis is important when looking at the inflammatory response which yields ROS, resulting in some microbicidal effect of TiO_2_ and improving the performance of biomedical implants. As there is a huge scientific community working on biomedical implants who are looking at improving the performance, regenerative ability, and other properties of Ti-based materials, discussions on Ti and Ti-based oxides for biomedical applications, the safety of these materials, and the inflammatory condition are also included. Finally, a conclusion/future perspective in terms of photo- and dark catalysis on Ti-based oxides for disinfection and bioimplant application is given.

## 2. TiO_2_ Photocatalysis

Since photocatalysis is mainly due to redox reactions of photogenerated charge carriers producing reactive surface species and that they mostly occur in the presence of water and/or oxygen, it is important to look at the role of reactive oxygen species (ROS).

### 2.1. Reactive Oxygen Species in TiO_2_ Photocatalysis

ROS can be considered primary intermediates of photocatalytic reactions with these four recognized as the main ones: hydroxyl radical (·OH), superoxide anion radical (·O_2_^−^), hydrogen peroxide (H_2_O_2_), and singlet oxygen (^1^O_2_) [57,59]. ROS seem to form mainly from the interaction of the VB hole with molecules (such as H_2_O) or species, oxidizing the latter and typically resulting in ·OH centers [58,60,61]. This could also happen in hole-trapping processes in TiO_2_, such as at bridging O_2_^−^, resulting in the formation mainly of ·O^−^ (“deprotonated ·OH”) [22,62,63]. Because of the high potential barrier of free ·OH for desorption, adsorbed ·OH is considered more favorable and is usually equated to trapped holes due to the adsorption–desorption equilibrium [22,57].

**Figure 2:**
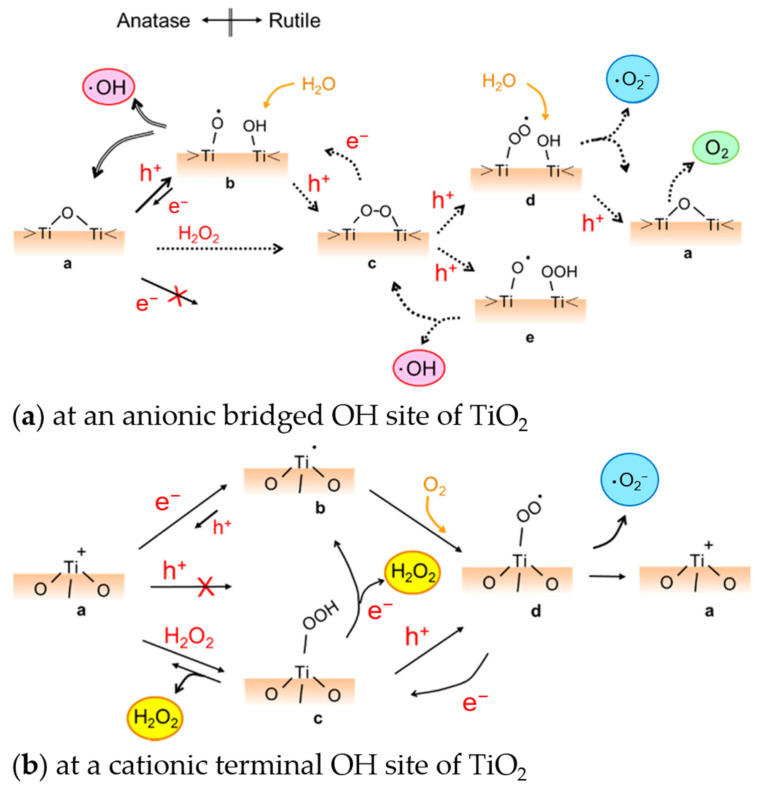
Photocatalytic reaction pathways on TiO_2_ proposed (**a**) at a bridge OH site and (**b**) at a terminal OH site. Reprinted (adapted) with permission from Nosaka, Y., Nosaka, A. Understanding Hydroxyl Radical (•OH) Generation Processes in Photocatalysis. *ACS Energy Lett.* **2016**, *1*, 356–359, doi:10.1021/acsenergylett.6b00174. Copyright 2016 American Chemical Society [58].

A detailed summary of generating the four major ROS on the TiO_2_ surface can be viewed in terms of bridging and terminal OH sites (Figure 2) [58]. The reactions occurring at the anatase and rutile are differentiated by the arrow lines (double lines are restricted to anatase), whereas broken lines refer to adsorption/desorption. At the bridged OH site (Figure 2a), a photogenerated hole attacks the O^2−^ bridge (step a), forming Ti-O· and Ti-OH (step b), which can be reversed by recombining with an electron from the CB. Some surface-trapped holes at the anatase can be released as ·OH into the solution. At the rutile surface with its suitable distance between adjacent Ti surface atoms, a different scenario occurs. Once another hole is formed in the same trapped hole-containing particle, the hole could migrate and interact with the existing hole resulting in a peroxo-bridged structure at the surface (step c). Further reactions of these structures could then generate other ROS [57,58].

At the terminal OH site (Figure 2b), a photogenerated electron can interact and is trapped at the Ti^4+^ site, transforming it to Ti^3+^ (step b) [64]. The trapped electron in the Ti^3+^ could then reduce oxygen to form an ·O_2_^−^ (adsorbed) (step d) (which, with further reduction, could become an adsorbed H_2_O_2_ or as (Ti)-OOH (step c)). The H_2_O_2_ that is adsorbed could also be reoxidized to produce an adsorbed ·O_2_^−^, which can be desorbed to return to the initial state (step a). As the peroxo bond needs to be dissociated, the production of ·OH is highly unlikely when the adsorbed H_2_O_2_ is being reduced [57,58]. These schemes (Figure 2) also show a sensible explanation for the influence of the adsorption of H_2_O_2_ in forming ROS, which has been well-considered for increasing the photocatalytic performance of TiO_2_.

Adsorbed ROS can also have a more direct impact on photocatalytic performance [57]. Anatase and rutile show different reactivity towards forming ·OH and ·O_2_^−^ [28,65], likely due to their H_2_O_2_ adsorption [63] in addition to their band edge alignment. One-step oxidation of H_2_O_2_ produces ·O_2_^−^, which is more remarkable for anatase, whereas one-step reduction produces ·OH, which is only observed for rutile or rutile-containing forms and is believed to be due to the structure of the adsorbed H_2_O_2_ on rutile vs. anatase [66].

### 2.2. Nanomorphologies and Structural States of TiO_2_

**Figure 3:**
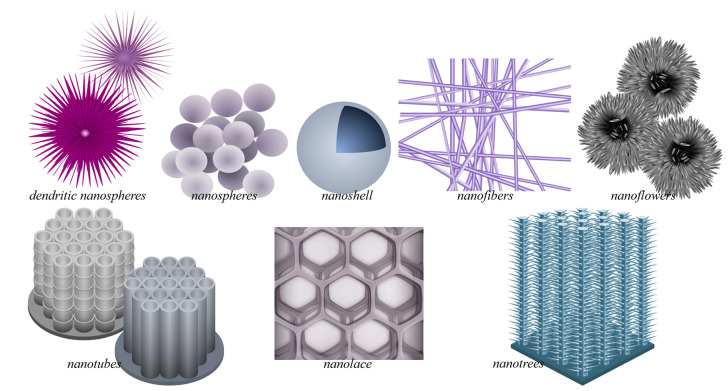
Plethora of TiO_2_ nanostructures. This includes free-standing nanomaterials, such as TiO_2_ dendritic nanospheres, nanospheres, nanoshells, nanofibers, and nanoflowers, and nanostructures grown on bulk substrates, such as nanotubes, nanolace, and nanotrees. Nanomaterials present increased catalytic activity due to their interesting properties (increased surface area, enhanced charge separation, light absorption/harvesting), which are desirable in catalytic TiO_2_ applications.

Due to its wide applicability, TiO_2_ has been produced via different means, with the resulting TiO_2_ structural polymorphs—i.e., anatase, rutile, or brookite, among others [67]—and morphology being highly influenced by the preparation method. The different TiO_2_ morphologies add to the variety of properties and performance exhibited by TiO_2_. In addition to bulk TiO_2_ [68,69,70,71], in recent decades, TiO_2_ nanomaterials of various morphologies have also been developed, resulting in the current plethora of TiO_2_ nanomorphologies (Figure 3). These have been synthesized using different means for various targeted applications, achieving a range of photocatalytic efficiencies (Table 1). Note that some morphologies are preferably prepared using certain procedures (e.g., sol-gel method for nanopowders and anodization for nanotubes), whereas some procedures (e.g. hydrothermal synthesis) can be used and modified to produce various morphologies (such as nanospindles, nanorhombus, nanorods, or nanosheets).

The morphologies of nanosized titanium dioxide can be grouped according to their dimension classifications: zero-dimensional (0D) includes nanopowders, nanocrystals, and quantum dots (QD); one-dimensional (1D) includes nanowires, nanofibers, nanotubes, and nanorods; two-dimensional (2D) includes nanosheets; and three-dimensional (3D) includes nanotube arrays. Mixed morphologies, such as nanosheets with QDs, also exist.

Many studies on photocatalysis have been conducted on nanoparticle suspensions [98,99,100], which are inconvenient and result in practical difficulties [8] because complete photocatalyst recovery is challenging. Therefore, studies have resorted to photocatalyst immobilization [101,102,103,104], which, however, requires catalysts of high activity. TiO_2_ of different nanomorphologies were used, evaluated, and/or compared in terms of performance [105,106,107], and some of these are in Table 1 to show the influence of morphology on photocatalysis. The photocatalytic performance reported, usually measured by dye degradation rate, varies and depends on the experimental setup/condition, such as the illumination and probe used, though the typical pseudo-first-order rate constant *k* is within the 10^−3^–10^−1^ min^−1^ range. Therefore, studies also sometimes include a reference TiO_2_, such as commercially available P25 for benchmarking. Nevertheless, from this summary (Table 1), one also sees that generally, some improvement in photocatalytic performance is brought about by nanomorphology based on the obtained *k* values being mainly in the 10^−1^–10^−2^ min^−1^ range for systems with varied morphology, which are at least one order of magnitude better than the usual for those with nanopowders (*k*~10^−2^–10^−3^ min^−1^).

Post-synthesis heat treatment (calcination/annealing) mainly dictates the structural state. Heat treatment can be performed to transform the amorphous state to rutile (≥600 °C) or anatase (300 °C to 500 °C) or to transform anatase to the more stable rutile [108]. The crystalline state also influences photocatalytic activity. It is commonly agreed that anatase is better than the other states (such as rutile) due to its higher surface affinity (i.e., better adsorption and probably due to the ROS formation as discussed in Section 2.1) and slower recombination rate. However, mixed states (such as the case of P25) also exhibit good photocatalytic activity, though the surface crystalline state seems to play a more crucial role in such cases of mixed states due to the fact that photocatalytic reactions take place at the surface [86].

#### 2.2.1. Safety of TiO_2_ Nanoparticles

Though TiO_2_ is considered a safe, biologically inert material [109,110], the development of TiO_2_ NPs with novel properties and applications resulted in its increased use and production. Hence, it has to be evaluated in terms of its toxicology. TiO_2_ NPs are posed as possible carcinogens to humans, though TiO_2_ is allowed for use as an additive (E171) in the food and pharmacy industry [111]. A sound basis of why TiO_2_ NPs have been scrutinized is the observed appearance of their unique size-dependent properties when inorganic NPs reach the limit of ≤30 nm in diameter. In this size range, drastic changes in the behavior of the NPs can appear, enhancing their reactivity at the surface [112]. While the increased reactivity at this size renders their enhanced catalytic effect, undesirable reactivity could also occur. The main adverse effects caused by TiO_2_ NPs seem to be due to their ability to induce oxidative stress, resulting in cellular dysfunction and inflammation, among others [113]. At high levels of oxidative stress, cell-damage responses are observed, whereas, at moderate levels, inflammatory responses may kick in due to the activation of ROS-sensitive signaling pathways [114,115].

TiO_2_ NP-induced oxidative stress is therefore related to increased formation of ROS and the resulting oxidized products and to the decrease in the cellular antioxidants. The damage and extent caused depend on the physicochemical properties of the titania particles. For instance, ·OH production depends on the TiO_2_ NP crystal structure and size and was found to correlate with cytotoxicity, e.g., against hamster ovary cells [116], pointing to ·OH as the main damaging species for UV-irradiated TiO_2_ NPs [117]. There are conflicting studies on whether it is the size or the irradiation of TiO_2_ that contributes to its ability to induce oxidative stress, and the readers are referred to extensive reviews, such as Skocaj et al.’s [118], on this topic and other TiO_2_ toxicity related discussions.

### 2.3. Photocatalytic Disinfection Using TiO_2_ Nanostructures

The photocatalytic ROS production on TiO_2_ NPs, while it may pose some health risks, can also provide benefits. As early as 1985, the photocatalytic microbicidal effect of TiO_2_ has been reported by Matsunaga et al. [119]. More studies have then been carried out on the bacteria-killing action of TiO_2_ [120,121,122,123]. Maness et al. [120] attribute this effect to the lipid peroxidation in the microbe (in their case, in *E. coli*) due to the photocatalytic oxidative property of TiO_2_ NPs. Upon initiation of lipid peroxidation by ROS, propagation can happen via the generation of peroxy radical intermediates, which can also react with other lipid molecules. Superoxide radical could also be involved, as it can also be photogenerated on TiO_2_. This can react with an intermediate hydroperoxide to form new reaction chains that can go through the damaged cell membrane. Once the cell wall is broken down, TiO_2_ NPs themselves could also possibly directly attack the cell membrane [120]. It is important to remember though that the microbe’s response to photocatalytic disinfection action can also be influenced by its level of protective enzymes against oxidative stress [122].

It is generally accepted that the photocatalytic antibacterial properties of TiO_2_ are due to its ROS formation, whereby ·OH is thought to play a crucial role [122]. Yet, novel TiO_2_-based materials also point to the role and use of other ROS. The development of different TiO_2_ nanostructures paves the way for advancements in photocatalytic disinfection on TiO_2_, and some examples, also in relation to the formation of ROS, are presented here.

**Figure 4:**
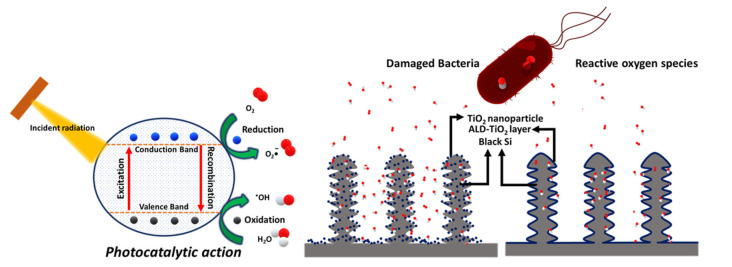
Antibacterial TiO_2_ nanoparticle hybrid nanostructures. The photocatalytic ROS generation on bacterial-disruptive nanostructures presents a low-cost antibacterial technology. Reprinted with permission from [124]. Copyright 2022, The Authors. Published by American Chemical Society. CC BY-NC-ND 4.0 license (https://creativecommons.org/licenses/by-nc-nd/4.0/).

Nanocomposites made from TiO_2_ NPs on Si nanostructured surfaces can be used as antibacterial surfaces for dental and orthopedic implants, and the TiO_2_ NPs themselves can be spray-coated to surfaces for disinfection of microbes upon irradiation [65] (Figure 4). The nanostructures are said to rupture the bacterial cell wall, whereas the ROS from TiO_2_ NPs can oxidize organic matter (such as bacteria) to prevent bacterial growth [124], which could eventually form biofilms. On the other hand, the free radicals photogenerated on TiO_2_ can also disrupt and destroy biofilms. This is important since killing bacteria within a biofilm is quite challenging; the biofilm shields the bacteria from antibiotics, antibodies, and immune cells [125,126]. Using TiO_2_ exposed to UVA, the biofilm formation of *P. aerigunosa* was inhibited via ROS attack to disrupt the bacterial cell membrane (disabling bacteria to form biofilm), and the ε-poly(L-lysine) of the cells already in the biofilm was weakened [65]. Through a plasma electrolytic oxidation process, Nagay and coworkers produced N- and Bi-doped TiO_2_ coatings that kill bacteria because of the generation of ROS upon visible light exposure [51,127].

Efforts to extend photosensitization with TiO_2_ were performed by forming composites with materials such as MoS_2_ and *l*-arginine and/or doping with Yb and Er [51,128]. However, the broader spectral range or near-IR sensitization is usually brought about by the additional component (and not by TiO_2_). On the other hand, other works utilize the photogenerated charge carriers from TiO_2_ and enhance the catalytic effect by the addition of other components for antibacterial purposes. For example, TiO_2_ combined with graphdiyne (GDY) was synthesized into nanofibers by electrostatic force to produce ROS and prolong the antibacterial effect. When exposed to light, electrons and holes are generated on the TiO_2_ and GDY surface, with the photogenerated electrons of TiO_2_ being easily transferrable to the GDY surface. There, ·OH and ·O^2−^ are formed because they can react easily with water and O_2_. The extended lifetimes of the charge carriers enhance ROS generation and the resulting bactericidal effect. Overall, these processes inhibit the methicillin-resistant *S. aureus* (MRSA) biofilm formation and promote the regeneration of bone tissues [51,65,129].

In general, the photocatalytic disinfection by TiO_2_ nanocomposite antimicrobial coatings entails the incorporation of inorganic metals/nonmetals (such as Ag, Cu, Mn, P, Ca, and F) and/or 2D materials (graphydiyne, MXenes, and metal–organic frameworks, etc.) into TiO_2_ to control the porosity of the surface, crystallinity, charge transfer, and disinfecting property against critical pathogens, such as *S. aureus* and *E. coli* but also H1N1, vesicular stomatitis virus (the safe surrogate virus for SARS-CoV-2), and the human coronavirus HCoV-NL63 [65]. Such light-catalyzed coatings could prevent microbes from reactivating to completely destroy them, and with the high mobility of ROS in the air, airborne microbes could also be targeted [130]. The high interest in the inactivation and disinfection of coronaviruses emerged recently due to the recent pandemic. Some of these studies were on the photocatalytic disinfection of coronavirus using TiO_2_ NP coatings, with the mechanism attributed to their generation of ROS [65,131,132]. For interest in antimicrobial coatings, readers are referred to Kumaravel et al. [65].

### 2.4. Efforts to Improve the Photocatalytic Efficiency of TiO_2_ Nanomaterials

As seen in Section 2.2, the photocatalytic activity of TiO_2_ nanomaterials improves by resorting to different morphologies. Their nanosize alone increases the surface area, providing more active sites. Additionally, due to the interesting properties afforded by its size, the use of nanomaterials also improves photocatalytic efficiency by enhancing charge separation and light harvesting and increasing the surface-to-bulk ratio. These improvements are presented in this section.

#### 2.4.1. Enhanced Charge Separation

The increased photocatalytic performance of TiO_2_ nanoparticles cannot be attributed to the increased specific surface area alone but also to the ***increase in the surface-to-bulk ratio*** with decreasing particle size. The latter results in shorter diffusion pathways that the charge carriers have to traverse to reach the surface, which is the photocatalytic reaction site [133]. Adding adsorbed species further provides electron/hole scavengers that could also improve the charge separation [133,134,135]. The decrease in the size though also blueshifts the TiO_2_ absorption edge [136] and could result in unstable NPs [137]. Therefore, an optimized size is needed to balance the properties in terms of charge separation, light absorption, and stability. During thermal treatment, which is typically needed for good crystallinity and increased photocatalytic performance, aggregation could occur, and this can be prevented by preparing highly-dispersed TiO_2_ clusters (such as those synthesized with zeolites [138]), which also shows high photocatalytic activity. These spatially separated TiO_2_ species were also prepared as single-site catalysts that also show high photocatalytic electron–hole pair reactivity and selectivity [22]. The high photocatalytic reactivity observed for highly dispersed TiO_2_ species is attributed to the highly selective formation of a longer-lived (up to µs), localized charge-transfer excited state compared to that of bulk TiO_2_ (ns) [133].

The aggregate formation is not always disadvantageous. The mechanisms portrayed for a single semiconductor NP (e.g., Figure 1, Figure 4, and Figure 5) are simplified, and a more accurate representation would consider that TiO_2_ NPs have the tendency to self-aggregate in aqueous solution to form a 3D framework. This happens by aligning their atomic planes with each other, allowing for efficient charge carrier transport without interfacial trap interferences in a so-called “antenna effect”. Through this, the photogenerated excitons in a nanoparticle will be transported throughout the network until they get trapped individually in a suitable site (e.g., via a redox reaction with an adsorbed electron acceptor/donor on one particle in the network). Charge carriers that are not trapped continue to traverse through the network until they themselves react. Therefore, through forming 3D aggregates, better electron mobility is achieved for TiO_2_ particles [22,139].

When the aggregates align, they act as if they are an array of nanowires that facilitate efficient CT throughout the network. In fact, 1D morphologies, such as TiO_2_ nanowires, nanotubes, and nanorods, are hailed for their efficient electron transport since the photoexcited charge carriers could move along the length, increasing delocalization and resulting in long diffusion lengths (>200 nm), which delays the charge recombination and prevents electrons from residing in traps [140,141,142,143,144,145]. For example, using TiO_2_ nanotube arrays (TNAs) for PEC water splitting can improve the photocatalytic efficiency and result in a photocurrent of up to 10 times since loss of photogenerated electrons is prevented with the electrons being able to diffuse along the tube towards the collecting substrate. The improved performance using TNA is said to not only come from enhanced charge separation and better electron transport due to the orderly arrangement [140,146,147,148] of this 3D structure but also due to better light harvesting [133,140], which is discussed in the next section.

Meanwhile, from Table 1, the photocatalytic activity of pure TiO_2_ nanomaterials is considerably small and requires mostly UV light due to its bandgap. Hence, various attempts were conducted to improve its efficiency and increase its absorption range. ***Doping*** is one widely used approach, and this has been performed with transition metal ions, such as Fe^3+^, which increased the efficiency for pollutant photodecomposition likely due to the fact that it decreases the *E_g_* and broadens the absorption range to the visible region [22,149]. Oxygen doping in TiO_2_ interstices can also improve photocatalytic performance by enhancing charge separation efficiency. For the photodegradation of methyl orange, O-doped or oxidative TiO_2_ showed a 2~3.7 times higher rate than pristine TiO_2_, with the former fabricated using KMnO_4_ to create trap sites to separate charges via bandgap impurity states [150].

***Depositing other catalysts*** has also been another approach. For example, a noble metal, such as Pt, Au, and Ru, can be deposited to increase the photocatalytic activity of TiO_2_ towards the decomposition of organics and photocatalytic water splitting [22,135,151,152,153]. This enhanced activity is likely due to improved charge separation as bulk electrons transfer to the metal and therefore to the surface of TiO_2_ [22,151,152,153,154]. Though not all photogenerated electrons transfer from the titania to the metal, the enhanced separation of photogenerated charge carriers increases the electron lifetime in TiO_2_. The separation is likely enhanced by the surface plasmon resonance (SPR) of the metal and the resulting increased localized EM field caused by the exposure of the metal to light. This induces charge carrier formation near the TiO_2_ surface, with which carriers can easily reach surface sites and improve charge separation. Loading TiO_2_ with gold instead of platinum also extends the absorption of TiO_2_ to the visible range up to near-infrared [22,155,156,157,158]. Using gold cores with TiO_2_ shells also exhibits remarkable photocatalytic activity compared to TiO_2_ due to enhanced separation of photogenerated charge carriers with the gold core serving as an electron trap [159].

In addition to metals, metal oxides can also be deposited on TiO_2_ to help improve charge separation and photocatalysis. For example, a Pd-NiO/TiO_2_ catalyst has been prepared to improve photocatalytic CO_2_ reduction. Due to the high electron density needed to drive this multielectron reduction, a p–n junction formed by introducing NiO to TiO_2_ helps to drive hole transport to NiO, whereas the Pd forming a Schottky junction with TiO_2_ facilitates semiconductor-to-metal electron transfer. These migrations towards NiO and Pd enhance the charge separation and result in high electron density around Pd, which can be used to transform CO_2_ efficiently and selectively to CH_4_ by reduction [160].

The coupling of the semiconductor to TiO_2_ can also be achieved by coupling it with another TiO_2_ phase. As mentioned before, mixed-phase TiO_2_ of anatase and rutile demonstrates improved photocatalytic performance than by using only pure anatase or rutile. This could be due to the formation of heterojunction when their valence and conduction band edges come into contact [65,161]. Several models were proposed to explain this synergistic effect in mixed phases (Figure 5). First was the model proposed by Bickley et al. based on the positions of the CBs of anatase and rutile in relation to each other [162]. Figure 5a (A) shows the model in which the electron transfer is from anatase to rutile. Separation of charges then happens in anatase and trapping of an electron occurs in the rutile phase. In another “spatial charge-separation model,” Hurum and coworkers (Figure 5a(B)) propose that the opposite is happening such that electrons are transferred from the rutile CB to a trapping site in anatase [163].

Meanwhile, the “interfacial model” proposed by Nair et al. looks at the band bending at the interface between anatase and rutile. The electron transfer should occur from the anatase to the rutile upon UV illumination due to the CB energy of the anatase being more negative than that of the rutile. When the illumination is with λ > 380 nm, the rutile is activated, and its CB shifts upward due to accumulated photoinduced electrons enabling the electrons in the rutile to reach the CB of anatase [133]. Thus, the “interfacial model” presents a directional movement of the electrons depending on whether the irradiation is ≤ or >380 nm and upon consideration of the interfacial band bending (Figure 5b) [164]. One can expect that the interfacial nanostructure plays a role in the electron transfer between the components and therefore in the overall photocatalytic performance. Further discussion on the advantages of mixed-phase TiO_2_ for photocatalysis can be found in the literature [133].

Fe_2_O_3_ has also been combined with TiO_2_ via photodeposition to enhance charge separation for contaminant decomposition and PEC water splitting. The achieved enhancement of more than 200% in complete mineralization kinetics was ascribed to the transfer of photoelectrons from the TiO_2_ to the Fe_2_O_3_, which in turn favors the rate-determining step of oxygen reduction [165]. Graphitic nanocarbon has also been added to TiO_2_ nanomaterials to improve charge separation. By covering short single-wall carbon nanotubes (SWCNT) (~125 nm) around TiO_2_ NPs (100 nm) using a hydration-condensation technique, longer lifetimes of photogenerated charge carrier and improved photocatalytic activity for the degradation of an aldehyde was achieved. This was better than nanographene and longer SWCNT hybrid systems. The shorter SWCNT provides greater interfacial contact with each TiO_2_ NP, more electron transport channels, and more efficient shuttling of electrons from TiO_2_ NP to SWCNT, delaying charge recombination. Improved SWCNT debundling with the short ones also affords these advantages to a larger portion of the composite [166].

The semiconductor junction can also be quite complex, involving materials such as MXenes. For example, Biswal and coworkers designed a Ti_3_C_2_/N, S-TiO_2_/g-C_3_N_4_ heterojunction to boost the spatial separation of charges and their transport in light of a photocatalytic water-splitting application [166]. This heterostructure was produced by thermal annealing and ultrasonic-assisted impregnation for H_2_ production that is up to ~4-fold higher than pristine S-doped titania. The dual heterojunction formed (a n–n heterojunction with a Schottky junction) likely not only enables effective charge carrier separation as CT channels [166] but also reduces the band gap due to the adjustment of the energy bands. In(OH)_3_-TiO_2_ heterostructures were also formed for enhanced photocatalytic H_2_ evolution. The band-gap tuning and improved charge separation resulted in up to a >15-fold increase in activity compared to commercial P25 [167].

#### 2.4.2. Enhanced Light Harvesting

The morphology or nanostructure of TiO_2_ also improves photocatalytic efficiency due to enhanced light harvesting. As mentioned above, nanoparticle aggregation (~500 nm) can improve light harvesting due to its high scattering effect, resulting in photon reabsorption. This increased visible-range absorption in turn increases the number of excited charges as seen in the increased current density. Such aggregates display unsmooth surfaces, resulting in better molecule adsorption in large surface areas and pore sizes [168].

***Nanotextured TiO_2_*** substrate produced by nanomolding also displays efficient light harvesting. The hierarchical nanopattern of dual-scale nanoscale craters featuring smaller bumps couples both the longer and shorter wavelengths of light resulting in a light trapping effect for efficient light utilization and at a wide angular range [169]. Similarly, cicada-wing-like structures were used as imprints to form nanohole structures of TiO_2_ decorated with Ag NPs (10−25 nm) for methylene blue (MB) photocatalytic degradation. The structure did not only exhibit extended absorption to the visible range but also greater light absorption, likely due to the SPR effect from Ag and the nanotexture of TiO_2_. This is based on the photocatalytic decomposition rate obtained for the Ag-TiO_2_ nanotexture being 2.7 times higher than the nanotextured TiO_2_ alone but more than 7 times higher than P25. This shows that even with just nanotextured TiO_2_, improved photocatalytic performance can be seen. As discussed in the previous section, the Schottky barrier formed between Ag and TiO_2_ could also improve the charge separation [170]. Hollow particles of ***TiO_2_ decorated*** with Au@Ag core–shell NPs also display enhanced light harvesting due to the combined strengths of the components of having a strong, broadened localized SPR, large specific surface area, and favorable light scattering properties [171].

***Orderly arrays of nanostructures***, such as TNA, serve as effective light scattering layers according to the Mie scattering theory. The Mie scattering effect displayed by anodized TNA or NPs has been used in solar cells to harvest more sunlight and enhance charge conduction [172]. Mie scattering is important in explaining particle size-dependent Raman enhancement observed with semiconductors [173,174] and is brought about by the plasmon resonance at the surface of the sphere causing signal enhancement that depends on the size as one comes closer to the lowest transverse electric mode of NPs. In addition to the Mie effect, size quantization also affects the Raman intensity obtained on TiO_2_ NPs [175].

**Figure 6:**
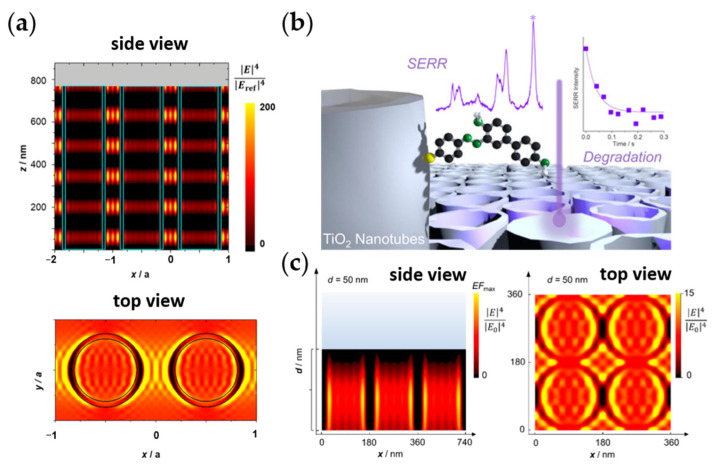
Electromagnetic (EM) field enhancement studies on Ti-based nanotubular arrays: (**a**) calculated EM field enhancement for the high aspect-ratio TiO_2_ nanotube array (TNA). The side and top view of the calculation show the distribution of the localized field hot spots for 3 and 2 tubes, respectively. The enhancement factor (EF) scale bar is shown at the left of the side view image. Adapted with permission from [50]. Copyright 2018 Wiley-VCH Verlag GmbH and Co. KGaA, Weinheim. (**b**) Photocatalytic azo-dye degradation on TNA correlates with EM enhancement. The inset shows the surface-enhanced resonance Raman (SERR) spectra of the adsorbed azo dye on TNA of high EM field enhancement and the corresponding exponential fit of the decay rate of the SERR intensity of the dye peak (marked by *). Adapted with permission from ref. [176]. Copyright 2019, the authors. Published by Wiley-VCH Verlag GmbH and Co. KGaA. Open access article. CC BY 4.0 license (https://creativecommons.org/licenses/by/4.0/); (**c**) EM field enhancement calculations for the partially-collapsed nanotube structure of TiN from nitridated TNA. The side and top view of the calculation show the distribution of the localized field hot spots for 3 and 4 tubes, respectively. The EF scale bars are shown at the left of the images. Adapted with permission from [177]. Copyright 2022 by the authors. Licensee MDPI, Basel, Switzerland. This article is an open-access article distributed under the terms and conditions of the Creative Commons Attribution (CC BY) license (https://creativecommons.org/licenses/by/4.0/).

The surface-enhanced Raman (SER) effect on semiconductors has also been well-observed [40,41,43,44,45], and the influence of nanostructuring on SER scattering, in particular on TiO_2_, has been investigated. Whereas CT and chemical contribution can provide an enhancement factor (EF) of ~10^3^ [46,47], EM enhancement is also afforded in TiO_2_ nanostructures of a high aspect ratio, such as nanotubes and nanofibers [42,48,49,50]. Han et al. [49] showed concrete evidence of morphology-dependent EM enhancement using cyt b_5_ heme as an indirectly-attached SER probe to reduce the chemical contribution to the Raman signals. Using EM field calculations, the particle’s aspect ratio was shown to increase the “hot spots” (regions of enhanced EM field) at the TiO_2_-water interface [49], improving the structure’s light-harvesting ability. Hence, other morphologies of higher anisotropy, such as TiO_2_ nanotubes, were further studied, showing a similar morphology-dependent EM field enhancement [42,50] (Figure 6). TNAs were shown to exhibit different Raman enhancements depending on the tube length, which fits the EM field calculation showing hot spots along the nanotube length [42,50] (Figure 6a). The TNA of high EM field enhancement was shown to perform better as a photocatalyst for visible-light-degradation of an azo dye pollutant immobilized on TNA (Figure 6b). Interestingly, the TNA’s optical response (i.e., its EM field enhancement) correlates with the photocatalytic degradation rate occurring on it [176].

Similar to other TiO_2_ nanostructures (see above), incorporating other components to form nanocomposites with TiO_2_ nanotubes can further improve not only the charge separation but also the light-harvesting ability. For example, S-doping or the addition of CdS NPs to TiO_2_ nanotubes resulted in enhanced visible-light water splitting [178,179,180]. Ultrafine Pt NPs were also added into TiO_2_ nanotubes for the efficient photocatalytic formation of methane from carbon dioxide and water. The nanotubes allowed for a homogeneous distribution of Pt NPs, which accept electrons and become sites for reduction, thereby also allowing efficient separation of charges [133]. Furthermore, even structures obtained from TNA somehow retain the light enhancement afforded by the 2D periodic arrangement of the nanotubes (Figure 6c). For example, nitridation of the TNA resulting in a partially collapsed nanotube structure of TiN also shows wavelength-dependent EM field enhancement and corresponding light enhancement [177].

The 2D periodic arrangement in TNAs enables them to behave as photonic crystals—with photonic lattices reflecting the light of certain wavelengths—bringing about localized EM field enhancement [42,50,181,182,183,184]. Interestingly, this photonic crystal-like character has also been observed in inverse-opal (IO) structures, which also achieved SER EF of around 10^4^ (though likely due to both chemical and EM contributions) [42,50,181], and which can also be made from TiO_2_. IO TiO_2_ also shows promising performance as photocatalysts [185,186,187,188], with their light harvesting extended to the visible range [187,188] and their ability to generate slow photons [188,189,190]. The slow photons have been shown to significantly increase the interaction of TiO_2_ with light and can work synergistically to amplify the chemical enhancement in the catalyst [186].

#### 2.4.3. Black TiO_2_

The photocatalytic efficiency of TiO_2_ nanomaterials can be improved by enhancing the separation of their charge carriers and improving their light harvesting and absorption properties (Section 2.4.1 and Section 2.4.2). Therefore, having a material that encompasses both is an ultimate surface-engineering achievement in this regard. Black TiO_2_ ticks both requirements and reasonably has then become a hot topic in TiO_2_ photocatalysis in the last decade or so.

Though previous studies already describe a similar material, as indicated in reviews [191,192,193], all papers seem to point to the work of Chen et al. [194] for introducing (and coining the term) “black TiO_2_” to describe the partially hydrogenated titania nanocrystals which exhibit a reduced bandgap due to a disordered layer at the surface of its crystals. This material exhibited a redshifted absorption onset to near-infrared (compared to the starting TiO_2_ nanomaterial), which was not surprising considering its visible color change. That is, due to the hydrogenation process, the crystals changed from white to black (hence the name). Consequently, this also results in a decreased bandgap of ~2.18 eV, making black TiO_2_ a good catalyst for visible-light irradiation. Additionally, it also exhibits good stability, making it an ideal catalyst for use under continued irradiation. From calculations, it also presents localized photogenerated charge carriers, indicative of slowed-down recombination, which is beneficial for photocatalysis. This makes the work of Chen et al. the first reported use of black titania for photocatalytic purposes [192].

From then on, many studies have been carried out to synthesize, characterize, and evaluate the photocatalytic performance of black TiO_2_ nanomaterials. Different methods have been developed to reduce TiO_2_ without the use of high pressure, as was conducted in the work of Chen et al. [194]. These include (electro-)chemical reduction [195,196], solvothermal hydrogenation [197], thermal reduction [198], reduction at the solid phase (reductant + heat) [199], anodization (and annealing) [200], ultrasonication [201] plasma treatment [202], gel combustion [203], or a combination of these [204]. Most of these strategies are similar to the synthesis of TiO_2_ nanomaterials presented in Table 1, with a reductant source/ reducing condition (either chemical, thermal, hydrogen, or reducing gases, such as hydrogen, nitrogen, and argon) added. Since black TiO_2_ is formed by the reduction of TiO_2_, it is also called “reduced TiO_2_” [205,206,207] or “hydrogenated TiO_2_” [208] and represented with the formula TiO_2−x_, the −x indicating the formation of oxygen vacancies [205,206].

What is interesting then is the concept of forming a novel material due to the introduction of surface defects, and yet, as this is also a TiO_2_ nanomaterial, it can also exist in different morphologies and structural states, resulting in a plethora of black TiO_2_ of various properties and photocatalytic performance. Table 2 gives examples of these materials and their photocatalytic performance in terms of organic compound degradation and hydrogen generation, with the latter being an important solar-driven application of black TiO_2._ Chen et al. [193] give a comprehensive review of black TiO_2_ nanomaterials, including their properties and examples of application, whereas Naldoni et al. [192] give a good summary of the photocatalytic H_2_ generation on black TiO_2_. The readers are encouraged to take a look at these reviews.

Some examples included in Table 2 are of different color naming (termed “colored TiO_2_”), such as green, grey, and blue TiO_2_ [192,195,208,210,211]_._ This is based on the understanding that the visual colors exhibited by TiO_2_ are brought about by intrinsic defects, such as due to the presence of Ti^3+^ and/or oxygen vacancies [192,195,200,208,211] or by doping with impurities [192,202]. Such defects create extra electronic states in the TiO_2_ bandgap, i.e., intraband gap states, which alters the optoelectronic properties of TiO_2_ [192]. Whether this is also the case for the color of black TiO_2_ is still a controversy. While some reports claim that the formation of these color-inducing intrinsic defects in TiO_2_ results from the hydrogenation [205], with the color depending on the extent of reduction and reducing condition [208], others propose that the black color is due to the disordered surface [194,202]. The disordered surface is caused by hydrogen and allows hydrogen to swiftly navigate around and induce electronic structural changes [212]. Midgap band states are formed because of the changes in the structure [203] brought about by the excessive lattice disorder. They can form an extended energy state by overlapping with the edge of the conduction band and also possibly combining with the valence band [202].

An effort to further unravel the relationship between the defect nature and photocatalytic activity of reduced TiO_2_ was performed by Will et al. [208] by considering that the intrinsic defects created on the surface are pertinent to the photocatalytic process and the location of the defect depends on the structural state and reducing conditions. They found that the introduction of Ti^3+^ at the surface results in a surface with substoichiometry, which activates the surface for photocatalysis. However, too long hydrogenation or too much Ti^3+^ is detrimental to its activity, as these provide additional recombination sites or prevent efficient interfacial CT. Surface roughness and strain were also not important for the activation of photocatalysis.

The photocatalytic activity of black TiO_2_ nanomaterial can be further enhanced by forming appropriate heterojunctions with other materials [208], a similar strategy used with TiO_2_ nanomaterials. Further, amorphous black TiO_2_ can also be synthesized and used for photocatalysis [201], which is important for applications such as for bone implants. Black TiO_2_ was shown to exhibit biocompatibility [213], regenerative properties [214], photothermal properties [213,214,215], and microbicidal action [213,215,216,217], among others. As such, black TiO_2_ shows promise for cancer treatment [214,215], as a bone implant coating, and for disinfection purposes [213,215,216,217]. Similar to TiO_2_, the photo(electro)catalytic disinfection with black TiO_2_ nanomaterials is also claimed to occur due to ROS, in particular, superoxide and/or hydroxyl radicals [216,217]. Nevertheless, the biosafety of black TiO_2_ needs to be further studied to intensify its application in the biomedical field.

## 3. Dark Catalysis on Ti-Based Oxides

In addition to photocatalysis, TiO_2,_ and Ti-based oxides also manifest “dark catalysis”. Here, we refer to dark catalysis in the context of the catalytic activity observed in the absence of irradiation. Early works on dark catalysis on TiO_2_ seem to have stemmed from the wide use of Ti alloys for biomedical applications. Due to the superb biocompatibility and good mechanical strength of Ti and Ti-based alloys, they are used as bone and dental implants. Thus, an interest in understanding the influence of Ti implants on the inflammatory response led to studies that looked at the Ti-H_2_O_2_ system in the dark. Ambiguities in the results of photocatalytic studies on whether the observed catalytic effect was brought about by light irradiation or by nanoparticle size also contribute to the catalytic effect observed in the dark.

As early as 1989, there was an interest in the influence of implants on the inflammatory response [218,219]. The role of Ti in Fenton-type reactions was examined [219,220] and thought to occur during the inflammatory condition. Ellipsometry studies showed that in the presence of H_2_O_2_, metals such as Ti readily oxidize to form metal hydroxides or metal oxides such that the body mainly interacts with the oxidized Ti instead of the bare metal [219]. Further, in the dark, TiO_2_ is found to catalyze the H_2_O_2_ decomposition based on observed oxygen evolution, though it is thought to unlikely occur through ·OH radicals [218]. The latter is based on ESR and spectroscopic results showing low ·OH formation for the Ti-H_2_O_2_ system in the dark [219]. When a Ti(IV)-H_2_O_2_ complex coordinates to a H_2_O_2_, a TiOOH matrix can be formed on the surface. This matrix can trap superoxide radicals, making the Ti(III) (reduced from Ti(IV)) likely inaccessible [218,219].

**Figure 7:**
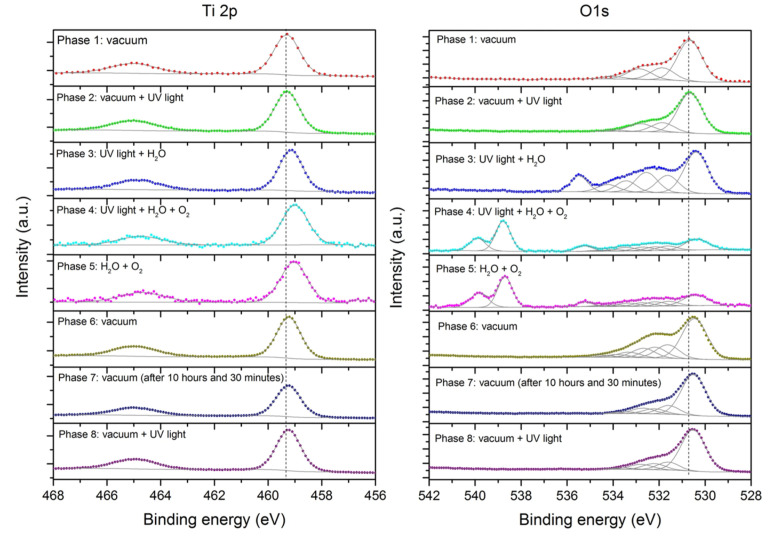
X-ray photoelectron Ti 2p and O 1s spectra of TiO_2_ exposed and unexposed step-wise to UV light, water, and oxygen. Phase 5 shows the presence of peroxides and dissolved H_2_O_2_ indicative of residual catalysis. Reprinted from ref. [228]. Copyright 2017, the author(s). Published by Springer Nature. This is an open-access article distributed under the terms of the Creative Commons CC BY license (https://creativecommons.org/licenses/by/4.0/).

The addition of H_2_O_2_ could also effectively promote the catalytic activity of TiO_2_ [220,221,222,223,224,225,226,227]. In Section 2, H_2_O_2_ and peroxides play a role in the photocatalysis of TiO_2_ (in the presence of UV, water, and oxygen). Once produced, peroxides and H_2_O_2_ can perform dark catalysis on TiO_2_. Such were the findings of Krishnan et al. in their investigation of the changes in the surface of photocatalytic bulk TiO_2_ powder in terms of UV exposure, as well as the presence of water vapor and O_2_ [228]. Using advanced XPS, changes in Ti 2p, O 1s, and the bridging and terminal OH were investigated. Maintaining the TiO_2_ activation state for a certain period in the dark was found to require the presence of water vapor and oxygen. The prolonged oxidative capacity of the TiO_2_ powder in the dark was ascribed to the appearance of peroxides and dissolved H_2_O_2_. Though the highest catalytic activity was observed in the combined presence of UV, water vapor, and oxygen, the nonreversal behavior of the XPS spectra upon UV light removal (Figure 7, Phase 5) points to continued TiO_2_ activity and indicates that the presence of H_2_O and O_2_ is enough to retain the dark catalytic activity for a time period (of around ⅓ of the duration when all three factors were present) [228]. Though this may seem to be only due to the residual effect from photocatalysis, the fact that prolonged and sustained catalysis even after removal of the light source continued and produced ROS points to the need to understand what is happening during this time. Understanding the continued catalysis in the dark will enable us to further exploit the advantages of this process.

Parallel to inflammation studies for biomedical implants, dye decomposition using TiO_2_ for purposes such as water treatment also continued to develop to extend the light beyond the UV range—towards visible light, ambient light, and even in the dark. Hence, from this field, an interest in dark catalysis on TiO_2_ has also developed. One of these works is on the bleaching of MB in the presence of TiO_2_ and H_2_O_2_ by Randorn et al. [220]. Even in the absence of light, they observed that catalytic degradation on TiO_2_ could occur, which was better on hydrated TiO_2_ (a hydrated amorphous TiO_2_ with a high surface area) than on Degussa P25. They noted that the mechanism could be different from photocatalysis with photogenerated charge carriers and instead must involve *Ti*^3+^/*Ti*^4+^ in a Fenton-reaction-like superoxide-driven process, whereby *H*_2_*O*_2_ is consumed directly:

(1) Ti3+(s)+H2O2→ O ·H+OH−+Ti4+(s)

where (*s*) indicates that the metal ions are from dangling bonds at the solid surface [220]. However, the catalytic effect in the dark observed in this work cannot be unambiguously distinguished from the surface adsorption effect in bleaching.

Sanchez et al. used a suspension of TiO_2_ and H_2_O_2_ to degrade MB in the dark and found that the TiO_2_ surface area and the concentration of H_2_O_2_ are crucial in catalysis. Using ESR, they found that free radicals are present in the mixture in the dark and attributed the observed catalysis mainly to ·OH and hydroperoxyl radicals (HO_2_·) [224]. The presence of the HO_2_·, together with other ROS (superoxide and hydroxyl radicals), was also detected by ESR in the work of Wiedmer et al. [226] in which MB degradation was performed nonirradiated on TiO_2_ (micro- and) nanoparticles with (3 vol.%) H_2_O_2_. The ·O_2_^−^/·OOH radicals seem to play a significant role in the dye degradation, as these radicals are present for those that show high dye degradation even if ·OH is more energetically favorable on anatase and is the most reactive among these oxygen-centered radicals. On the other hand, Zhang et al. [225] attribute the improved performance of the TiO_2_-H_2_O_2_ mainly to ·OH formation. In their work, ·OH (E^0^ = 2.80 eV) radicals can be formed by using facet- and defect-engineered TiO_2_ to heterogeneously activate H_2_O_2_ (E^0^ = 1.78 eV) into a defect-centered mechanism for Fenton-like catalysis. This involves surface Ti^3+^. The Ti^3+^ donates electrons to the H_2_O_2_ and generates ·O_2_^−^/·OOH and ·OH in the process [221,225].

Facets also have an influence on (photo-) and dark catalysis on TiO_2_. For example, (001) is considered the most reactive facet in anatase, likely due to its very high anisotropic stress. The surface reconstructs to reduce this stress by forming ridged atoms in every fourth unit cell and likely by the creation of ridge atom vacancies [229], which can interact with charge carriers and ROS.

Wei et al. showed that TiO_2_ (B) nanosheets and H_2_O_2_ can degrade dye molecules in the dark, though the process is accelerated in the presence of visible light or heat. They attributed this catalytic activity to the reaction of the nanosheets with H_2_O_2_ which can generate superoxide radicals [223]. Jose et al. attributed the dark catalytic H_2_O_2_ decomposition with hydrogen titanate nanotubes to occur primarily by generating and attacking ·O_2_^−^ rather than the hydroxyl radical [230]. Using (delaminated) titanate nanosheets, efficient removal of high concentrations of dyes at a wide range of pH can be achieved. The mechanism of this non-light-driven catalytic degradation involves the formation of the yellow complex surface Ti-OOH, the key species to strongly oxidize and degrade organic dyes into smaller molecules. Initially, H_2_O_2_ adsorbs and is followed by an exchange with Ti-OH groups at the surface [227]. In effect, the active species observed in the said work can be thought of as a Ti-coordinated hydroperoxyl unit.

The enhancement of the TiO_2_ catalysis in the dark upon H_2_O_2_ addition is due to the formation of ROS on the surface, including ·OH and ·O_2_^−^/·OOH. Wu et al. investigated the mechanism of this process by using TiO_2_ NPs with single-electron-trapped oxygen vacancy (SETOV). SETOVs are common TiO_2_ intrinsic defects. In the presence of H_2_O_2_, TiO_2_ with SETOVs can efficiently degrade organic dyes catalytically in the dark [221]. Using XPS and ESR, they found that SETOV mainly activates H_2_O_2_ in the dark by a direct contribution of electrons, which, in the process, forms both ·O_2_^−^/·OOH and ·OH to enhance the system’s catalytic activity. The steps in the mechanism for the dark catalytic ROS generation are given in Table 3.

Oxygen vacancies in general are said to play a pertinent role in dark catalysis. Such is the case for the decomposition of N_2_O on anatase (001) and (101), which, during the reaction, involves filling the vacancy [192,229]. The concentration of oxygen vacancies can be increased by calcination at a higher temperature. The oxygen vacancies reductively interact with oxygen to form O_2_^−^, which can increase the current density (for Hg^2+^ reactions, for example) on TiO_2_ [229,231].

In terms of biomedical applications, there is also growing evidence that ROS formation and its adverse effects are induced in the presence of TiO_2_ even in the absence of light. Unexposed anatase NPs induced higher levels of ROS within human hepatoma cells compared to unexposed rutile, with the former causing oxidative DNA damage [118,223]. DNA oxidative damage seems to be only brought about by nanoparticles as with ordinary TiO_2_ particles, without irradiation, cell survival was not affected (though the number of DNA strand breaks was also increased) [118,232].

### Microbicidal Effect of TiO_2_ in the Dark

A similar discussion to Section 2.3 is presented here but for the disinfection with TiO_2_ nanostructures in the dark. This is useful for certain TiO_2_ applications, such as with implants that will be in the dark after surgery. To prevent inflammation, strategies include ensuring the implant material surface has antimicrobial properties. As titanium naturally grows oxide and may be induced to grow thicker and more stable oxide films (see below), some natural bactericidal effect is also already afforded on Ti and its alloys. This is important since it is almost impossible to achieve a completely bacteria-free environment for surgery as most operating rooms are contaminated quickly and easily [36,37,233]. To highlight the catalytic effect of TiO_2_ on antibacterial action for biomedical implants, the discussion here is limited to the bactericidal effect of TiO_2_. Therefore, readers interested in strategies to improve antibacterial properties on Ti implants are referred to other reviews which have already summarized such strategies [234,235,236,237].

The microbial-killing action of TiO_2_ in the dark has been observed and recognized for a long time. Matsunaga et al. [119] observed that even in the absence of irradiation, ~10−12% of the *S. cerevisiae* cells were killed. A decrease in the colony-forming units of *S. sobrinus* with TiO_2_ in the dark was also seen by Saito et al. [121]. Other works then followed, mainly on photocatalytic disinfection with TiO_2_, in which the bactericidal effect of TiO_2_ in the dark was observed and recognized [120,122]. However, these works point to the disinfection effect in the dark that is residual from the bactericidal phototreatment. This effect is similar to and has been pointed out in the work of Krishnan et al. (see Section 3) [228]. They pointed out that the long-lived reactivity of TiO_2_ in the dark could explain the observed extended bactericidal effect of TiO_2_ in the dark. Indeed, Rincón and Pulgarin [238] also attribute this “residual disinfection effect” to the photoinduced generation of ROS that damaged and continued to kill bacteria in the dark [122,238]. These studies point to the fact that light may be needed for initiation but may not be continuously necessary for various applications [37,228], which is a beneficial finding for TiO_2_-based biomedical applications in relation to presurgical irradiated disinfection. In addition to ROS generation, TiO_2_ is also claimed to display bactericidal and self-sterilizing effects by altering the material’s surface free energy and electrostatic interaction with the microbial cell wall [65]. Moreover, as discussed in the previous section, ROS generation seems to occur not only due to photocatalysis but also in the absence of light.

Black TiO_2_, on the other hand, shows an electrochemical (EC) microbicidal effect which could be of the same disinfection rate as the photocatalytic effect [213]. Though radicals were not seen in ESR in the dark on black TiO_2_, in comparison to PEC treatment, the EC microbicidal result indicates that light is not necessary for black TiO_2_ to display microbicidal action.

## 4. Ti and Ti-Based Oxides for Biomedical Implants

**Figure 8:**
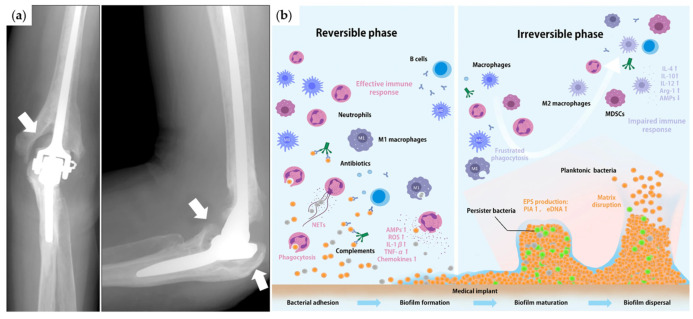
Challenges for bone (and dental) implants: (**a**) aseptic loosening of the implant commonly occurring due to “stress shielding”. Reprinted with permission from ref. [241]. Copyright 2019 by the Korean Orthopaedic Association. This is an open-access article distributed under the terms of the Creative Commons Attribution Noncommercial License (https://creativecommons.org/licenses/by-nc/4.0/); (**b**) occurrence of inflammation and biofilm formation. If osseointegration fails when host cells do not strongly attach to the implant, microbes can colonize the surface, leading to biofilm formation and inflammation. Reprinted (adapted) from ref. [38]. Copyright 2022, The Author(s). Published by Springer Nature. This is an open-access article distributed under the terms of the Creative Commons CC BY license (https://creativecommons.org/licenses/by/4.0/).

In the field of bioimplant application, one should also consider other aspects of the material for targeted implant usage. Ti and TiO_2_, for example, find applications in dental and bone implants due to their excellent biocompatibility and good mechanical strength. For bone implant application, for example, the material’s mechanical properties have an influence on the postimplantation healing of the affected bone area and the performance of the implant. When the material’s stiffness (Young’s modulus) is too high compared to the bone, the distribution of the postsurgical physiological load on the periprosthetic bone changes such that the implant handles more of the load, and the bone receives insufficient stress that it needs, i.e., “stress-shielding” occurs. This results in bone resorption, loss of density, and eventual atrophy, resulting in aseptic loosening causing implant failure (Figure 8a) [51,239,240,241]. Studies show that aseptic loosening accounts for at least 20–33% of orthopedic revisions due to implant failure [51,239,242].

Titanium and its commonly available alloys have a Young’s modulus of 100−150 GPa, which is still higher than that of bone (10−30 GPa) [1,243,244,245]. As such, efforts to reduce the alloys’ stiffness have been investigated. For example, β-type Ti alloys (which can be formed by adding stabilizers, such as Nb, Ta, V, or Mo) [243,246] can have a Young’s modulus of ~50−80 GPa and can even reach as low as 40 GPa when subjected to severe cold-working [1,29,30,243,244,247]. Ti displays nontoxicity high strength [245,248], which also has to be considered together with stiffness [1,29,31,244,245,246,247] (opposing in nature) when designing alloys for implant application. Together with these two, the corrosion properties should also be considered [1,29,31,243,245,249].

Corrosion response determines how the material behaves when exposed to the physiological electrolyte and environment during and post-implantation [29,30,31]. Ti and Ti-based alloys were developed mainly to improve the mechanical properties of implant materials, especially for load-bearing purposes by increasing the fatigue properties. Due to the thin, passive TiO_2_ film that develops on the surface, Ti and its alloys also display good corrosion properties. This thin TiO_2_ film is stable in natural and artificial physiological fluids [243], and elements added for alloying should therefore not disrupt the oxide layer formation. Nb and Zr, for example, can be added as alloying elements to Ti because their (mixed) oxides remain passive and contribute to corrosion resistance. A challenge alongside the oxide formation in alloys is in the case of uneven distribution of the elements in the different phases, unstable formation of the passive oxide film could occur and would lower the material’s resistance to corrosion [1,29,30,250]. All these aforementioned properties of Ti and its alloys, therefore, have to be considered for their advancement in use as modern implant materials. Further, when improving processability, such as in additive manufacturing or by adding components to achieve other functionalities, the influence of such modifications on the aforementioned properties has to be considered [1,29,30].

A critical functionality for bone implants is the formation of a robust and lasting attachment between the implant and the bone [251]. Therefore, efforts to increase the success rate of implants also entail improving bone adhesion and growth on the implant surface. This is an advantage for Ti alloys which are known to exhibit osseointegration, allowing for direct anchoring of the implant to the bone even though Ti is considered inert. Nowadays, the understanding of osseointegration considers the implant as a foreign body from which the body tries to defend and shield itself by forming bone tissues to surround the implant [39,243]. Effective implant osseointegration will not only promote healing but also prevent infection by not allowing pathogens and microbes to colonize the implant surface. This so-called *race for the surface* [34,35,36,37] determines whether the implant will succeed or fail, especially after surgery and during wound healing [34]. This depends on whether the host cells can attach to the implant irreversibly before bacterial cells do so in the irreversible phase of biofilm formation (Figure 8b) [38]. To further improve the interface between bone and the implant, biocompatible oxides, such as TiO_2_, are used to facilitate this bridging. The roughness of the surface contributes to the attachment dynamics displayed by the bacteria and the host cells towards the implant, making nanostructured TiO_2_ beneficial for such cases [36]. For further interest, the readers are referred to more extensive reviews on the surface modification for biomedical and antibacterial properties [51,52].

**Figure 9:**
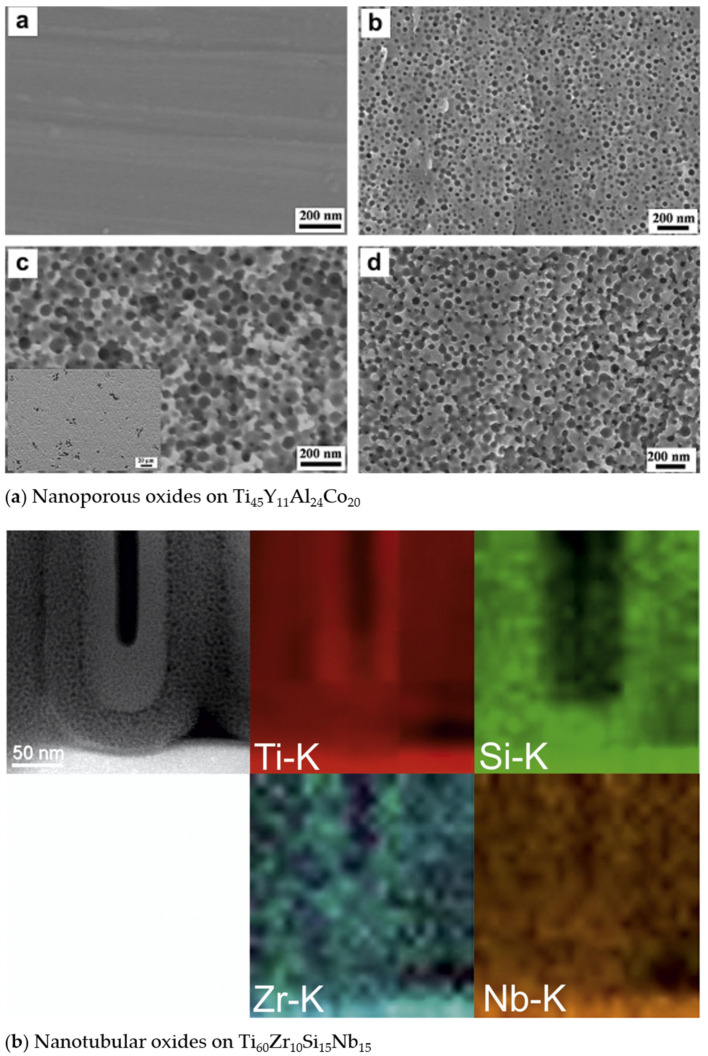
Nanostructured oxide layers grown on glassy alloys: (**a**) nanoporous oxides on a Ti-Y-Al-Co alloy. The electron microscope images show the nanopores formed after various treatments. Reprinted (adapted) with permission from ref. [33]. Copyright 2009 Elsevier Ltd.; (**b**) nanotubular oxides grown on Ti-Zr-Si-Nb alloy. Electron microscope images show the layered tubular structure and the distribution of the different alloying elements in the oxide. Reprinted (adapted) with permission from ref. [53]. Copyright 2016 Elsevier B.V.

While the implant surface is quite important for its successful osseointegration, the bioinert native TiO_2_ layer (2−5 nm) [53], however, does not allow the implant to bind easily and strongly with bone tissues. Further, this layer can be disrupted due to tribological factors (e.g., fretting) in the presence of fluoride (as for dental implants) or caused by oxidative stress brought about by highly aggressive ROS, such as when inflammation occurs due to the implantation process or during implant degradation (see Section 4.1). Because of the debilitating effect of the disruption of the thin native oxide film on Ti-based implants, efforts were done to produce thicker and more stable layers of Ti-based oxides to improve the materials’ surface bioactivity [53,54,55], favoring bone cell adhesion and proliferation and matrix mineralization promotion [31,56]. Direct oxidation of Ti implants by treatment with H_2_O_2_ or NaOH to induce the formation of a TiO_2_ layer can be performed to enhance the bioactive fixation of Ti-based implants [252]. Other efforts also include growing Ti-based nanoporous oxides [33] and nanotubular oxide layers [53] on glass-forming alloys, such as Ti-Y-Al-Co for the former and Ti-Zr-Si(-Nb) for the latter (Figure 9). Similar to other alloys, while the alloying elements are needed for the desired mechanical and corrosion properties for certain biomedical applications, mixed metal oxides could form. With the prospect of growing nanotubular layers, for example, the effect of the alloying elements on the tube dimensions should also be considered. These alloying elements can have different electrochemical oxidation rates and stabilities in the electrolyte [53]. Nb_2_O_5_, for instance, is more resistant in F^−^- induced dissolution compared to TiO_2_ [253]. Thus, Nb could slow down (or accelerate) the nanotube growth depending on the anodization electrolyte used, whereas Zr usually increases the nanotube length at the expense of the diameter growth. Such effects could result in two- (or multi-) scale diameters of the nanotubes [53]. Titanium alloys, such as Ti6Al4V, that were pretreated with H_2_O_2_ can also develop a relatively thick and porous surface layer, which could promote precipitation of hydroxycarbonate apatite to achieve a seamless transition between the peri-implant bone region and the implant materials, improving osseointegration [243].

### 4.1. Safety of Ti-Based Implants and Inflammation

In addition to the safety concern regarding titanium dioxide NPs [254] (also see Section 2.2.1), Ti-based materials, while generally considered safe, have also been increasingly scrutinized regarding their toxicity [114]. Extensive reviews on this, such as Kim et al.’s [114] exist, and when interested, the readers are encouraged to read them. The main concern regarding Ti as an implant material is the possibility of its degradation-induced debris formation and chronic accumulation. This can cause inflammation [118,255], such as perimucositis or peri-implantitis [118]. Further, these debris can also accumulate in the spleen, bone marrow, and liver and may result in systemic diseases and other health issues [114].

The degradation of Ti-based materials due to implantation results in the formation of a thick layer of TiO_2_ on the surface of the implant (initially determined from color change [256]), indicative of the occurrence of corrosion processes [243,256,257]. Evidence of material dissolution is also present. Such is the case for the β-phase of Ti6Al4V [258] in which its selective dissolution could originate from the attack of H_2_O_2_. Other evidences of Ti degradation including oxide growth within, oxide-induced stress corrosion cracking, and the presence of much-concerning periprosthetic debris have also been observed [243]. Alloys such as Ti-Al-V can also cause inflammation by inducing the release of mediators (prostaglandin E2, tumor necrosis factor, etc.) and may affect the periprosthetic tissues to cause osteolysis [114,255,259]. While the degradation of Ti-based materials could happen due to inflammation, implant deterioration could also be due to other factors which may be electrochemical, chemical, biological, and/or mechanical in nature [243].

Inflammation is the immune system’s response to detrimental stimuli involving white blood cells (leukocytes). This occurs, for instance, due to the wound or the presence of infectious species or foreign debris. The leukocytes respond by either engulfing the invaders (phagocytosis) or by increasing their O_2_ consumption to produce ROS [243,260,261]. Cell-signaling proteins are also produced by leukocytes to recruit more leukocytes, amplifying the process. When phagocytosis could not occur due to the size of the target (e.g., large implant debris), macrophages merge together to produce foreign body giant cells [243,262]. In the case of bones, these foreign body giant cells can be the osteoclasts (bone resorption cells), which can also form phagocytosis and produce ROS [243,263].

**Figure 10:**
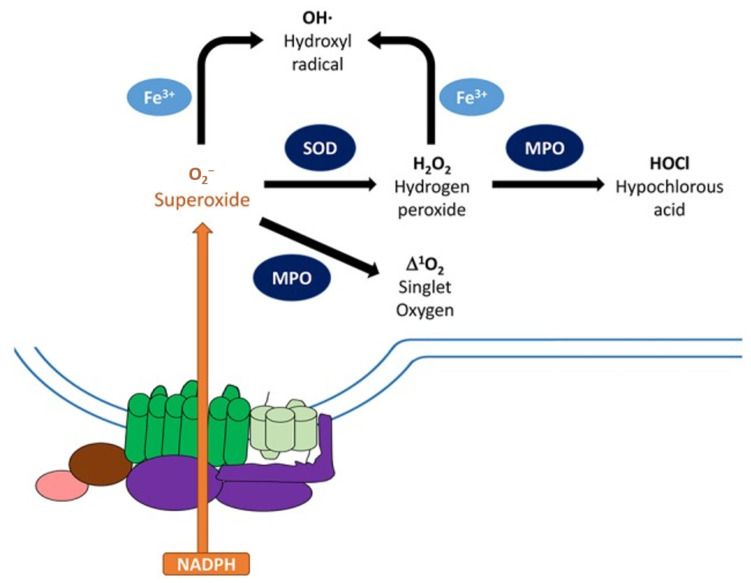
Biochemical reactions involved in reactive oxygen species (·O_2_^−^, ·OH, H_2_O_2_, singlet oxygen, and HOCl) formation during inflammation. Reproduced (adapted) with permission from [261]. Copyright 2017 Nguyen, Green, and Mecsas. This is an open-access article distributed under the terms of the Creative Commons Attribution License (CC BY) (https://creativecommons.org/licenses/by/4.0/).

At different phases of inflammation, ROS can be produced by specific enzymes, and the biochemical reactions involved are depicted in Figure 10 [243,261]. As H_2_O_2_ plays a key role in the inflammation process involving the immune system, it has been used extensively for in vitro corrosion studies in simulated inflammatory conditions. Based on the observed effects of inflammation, inflammatory studies are therefore carried out and evaluated by looking at the metal release, phase dissolution, and oxide formation on the material under evaluation [243]. Electrolytes closer to the physiological condition have also been used, whereby it was observed that a synergetic effect could occur with the presence of H_2_O_2_ and albumin in terms of metal release and material implant dissolution but not oxide layer growth (at least for Ti6Al4V) [114,243].

While inflammation can be useful, such as at the early stage (acute) needed to heal the wound and prevent peri-implant infection (duration ~1 week), inflammation that lasts for weeks or months (chronic) results in health issues and can generate pain, irreversible cell degradation or DNA damage, and implant damage [243]. Periprosthetic inflammation has been found to correlate with the increased level of Ti (whether dissolved or as particles) and could result in bone resorption of the surrounding region (in the case of peri-implantitis) [114,262]. Additionally, Ti exposure has also been related to the occurrence of yellow nail syndrome, wherein the person’s nail exhibits discoloration associated with sinus inflammation and coughing, among other symptoms [114,264,265,266]. In addition to Ti, considering its alloys, the other constituents could also result in health issues pertaining to those elements (Co, Cr, and Ni for instance have higher toxicity) [114,243].

## 5. Conclusions and Future Perspectives

Catalysis on TiO_2_ is mainly used and is more effective in the presence of light of sufficient energy (i.e., with *E* ≥ *E_g_*). Nowadays, with many modern forms of TiO_2_, including black TiO_2_, and advanced structures incorporating them, this can be extended to visible and IR light. This strategy (extending the absorption range or tuning *E_g_*) and other means to improve TiO_2_ photocatalysis remain relevant in furthering the applications which benefit from this field. Extensive knowledge of TiO_2_ photocatalytic mechanisms can be compared and contrasted to what is thus far understood regarding dark TiO_2_ catalysis. Both processes involve ROS generation; however, due to the absence of light needed for charge carrier generation in the case of dark catalysis, it seems that defects are crucial sources of charges to activate ROS formation. This may be the case with black (and colored) TiO_2_, whereby surface defects could further promote ROS generation and, consequently, (photo)catalytic activity. Thus, in terms of implant application, looking at both photo- and dark catalysis could give a more holistic overview as implants could be also exposed to light prior to implantation, resulting in the so-called residual disinfection effect.

The residual disinfection observed after the removal of irradiation can also be taken advantage of by developing strategies to address the growing concern about antibacterial resistance. Deepening the understanding of what is happening during residual catalysis could help design materials, processes, and strategies to address such challenges and prevent implant/device failure. For example, while there is a general understanding of the involvement of ROS, intracellular peroxidation, and the disruption and direct attack of TiO_2_ NPs themselves in this (photoinduced) residual bactericidal effect of TiO_2_ [122], further details on what is happening could be beneficial in obtaining a nuanced understanding to aid designing materials with improved bactericidal action. The specific mechanisms for each different microbial species also need to be figured out. As many of these microbial species evolve continuously, such as by developing into different strains, such mechanisms should also be updated regularly. The fact that the viability of bacteria differs when inside and outside a biofilm should also be considered. Though mechanisms of actions of TiO_2_ against bacteria outside and within a biofilm have been proposed [51,65,125,126,129], these understandings need to be further deepened.

In addition to photocatalysis and residual catalysis on TiO_2_, it is important to also explore and further establish TiO_2_ catalysis in the dark. Whereas over the past years, significant development has been carried out to unravel the dark ROS formation mechanism on TiO_2_, such as by looking at the role of SETOVs in dark activation of H_2_O_2_ on TiO_2_, the mechanism is yet to be confirmed (if possible) in non-SETOV-incorporated TiO_2_ nanostructures. The role of other intrinsic defects (Ti^3+^ and surface oxygen vacancies) on the dark catalysis of TiO_2_ should also be further studied, and a mechanism including these remains to be proposed. The role of extrinsic defects, e.g., for doped TiO_2_ structures, in dark catalysis also needs to be studied. This is important, especially when considering that Ti alloys include other elements which could introduce impurities to TiO_2_ or form mixed oxides with Ti. Further knowledge of dark catalysis on TiO_2_ can also be helpful in advancing the use of TiO_2_ for biomedical applications. Implants and devices will be in the dark after surgery and important in vivo processes to ultimately determine implantation failure or success, such as inflammation, tissue regeneration, and possibly antibacterial action around the implant also occurs in the absence of irradiation. Thus, the role of ROS in dark TiO_2_ catalysis in view of these processes is crucial in addressing the challenges in Ti-based implant application.

Understanding the dark catalysis on TiO_2_ in vitro can shed light on what could be happening with TiO_2_/Ti implants in vivo and help in rationally designing materials that could take advantage of ROS formation and/or catalysis for implant application. The sensitivity of ROS generation observed in several studies to factors such as pH and concentration, the presence/absence of oxygen, and other bio-/molecules points to a possibility of a different mechanism happening in the physiological condition and also during inflammation. In vitro mechanistic studies on these implant materials using physiological conditions should therefore be eventually extended to in vivo studies.

Black TiO_2_ nanomaterials also introduce new opportunities to widen the application of TiO_2_ in the biomedical field. The fact that it can be produced on amorphous TiO_2_ and on other Ti alloys without necessitating heat treatment makes it attractive for bioimplants—which could be made from amorphous alloys. Further, in vivo *(*/in situ) phototreatment could be made possible as the light absorbance of black TiO_2_ could be tuned to lie within the biological near-IR window. Its redshifted broadened absorbance allows for photothermal application in relation to implant use, which is beneficial for cancer treatment, photothermal antibacterial disinfection, and so on. However, its biosafety has to be confirmed.

The development of new materials, while it is useful and advances the field, also entails the need to be investigated in terms of their ROS generation mechanism. For example, the incorporation of other components to further improve other desired properties, surface treatments, and the use of alloys forming (supposedly mixed) surface oxides can have an influence on the dark catalytic properties; their mechanisms would also need to be studied. Novel materials for implant application will also result in the formation of new microstructures of corrosion products (e.g., the oxide layer), which will also need to be evaluated in terms of their vulnerability to ROS attack. This is considering the plethora of processing available, the nanostructures that can be formed, the incorporation of bulk and surface modification, and the possibility of protocol changes with the advancements in the medical field, such as in the fight against antibacterial resistance. There are also findings on other Ti alloys that cannot be explained by the current existing understanding, and this points to the possibility that they involve modifications in the mechanism known for pure TiO_2_ alone. Additionally, a number of applications using TiO_2_ make use of its amorphous state, such as for TiO_2_ grown on glassy alloys that are used in dental implants. Apart from using it as a control in crystallinity effect studies, this structural state seemed to have been forgotten, especially in terms of understanding its catalytic activity (if any) and properties and performance in the presence of ROS. This is also relevant when considering black TiO_2_, which can be grown as an amorphous material and possibly also on an amorphous material. It is also necessary to investigate the catalytic activity of black TiO_2_, both photocatalytic and in the dark, and now also PEC and EC processes (with an increasing number of studies presenting such as comparative results), in terms of ROS generation when relevant. Therefore, the understanding of TiO_2_ catalysis needs to be extended to (and maybe modified for) these materials, which will make the already complex question (of understanding dark ROS generation on titanium-oxide-based implants and addressing challenges in the biomedical field in light of TiO_2_ catalysis) a tad more complex.

## Figures and Tables

**Table 1:** Reported photocatalytic performance of some TiO_2_ nanomorphologies.

Morphology	Material Dimension	Crystal State	Synthetic Procedure	Photocatalytic Performance ^§^	Targeted Applications	Ref.
Commercially available Degussa P25 nanopowder	30–40 nm	anatase + rutile	either deposited as film or used as dispersion/colloid	Pseudo-first-order rate constant, *k* = 0.0085–0.012 min^−1^ AO7 [72,73], RhB [73,74] degradation; UV light, 100–200 mW HeCd laser with I = 60 or 100 mW cm^−2^, 254~325 nm	Dye-sensitized solar cells, etc.	[72,73,74]
Quantum dots	~4.7 nm	anatase	autoclave method (+heating)	~18% MO photodegradation* (Xe lamp with a glass filter, 400 nm cut off, 100 mW cm^−2^) [75]; *estimated *k* = 0.0033 min^−1^	Energy and environmental applications	[75,76]
Nanocrystals/nanopowder/nanoparticles (NPs)/ nanospheres	particle size: 8–10.2 nm [77,78], 14–18 nm [79,80], 19–23 nm [81]	anatase	sol-gel method + heat treatment	*k* = 0.002−0.036 min^−1^ (MB degradation) [78,79,81], *k* = 0.090–0.105 min^−1^ (MR degradation) [77] (UV light 250–625 W; max. ~250–368 nm); 20% (NOx degradation after 60 min; UV light-20 W, 287.5 nm) [80], *estimated *k*~0.0037 min^−1^ UV light 250–625 W; max. ~250–368 nm)	Pollutant degradation and self-cleaning [77,78,79,81], exhaust gas decomposition [80], energy storage [78]	[77,78,79,80,81]
Nanoporous shell (polyimide support)	2.7–2.8 nm pore size, 12~20 nm shell thickness,	anatase	in situ complexation hydrolysis	~80−95% degradation rate* (365 nm, 30-W UV lamp) *estimated *k* = 0.04–0.07 min^−1^	Air purification and water disinfection	[82]
Nanospindles	~190 nm in length; growth direction along [001]	anatase	hydrothermal synthesis	*k* = 0.0306 min^−1^ (RhB degradation; UV illum.; sim. sunlight AM 1.5 G filter, (100 mW/cm^2^))	Dye-sensitized solar cells, etc.	[74]
Nanowires	100 nm diameter × 800 µm	anatase	hydrothermal reaction, proton exchangem calcination	*k* = 0.0154 h^−1^ (0 0.000256 min^−1^) (phenol degradation in water; LED UV lamp (18 W); ca. 12 mW cm^−2^)	Photocatalysis, gas sensors, etc.	[83]
Nanowires/ nanobelts	15 nm diameter	anatase	hydrothermal growth + heat treatment	51.96%* (MO degradation; 350-W Xe lamp); *estimated *k*~0.0066 min^−1^	Organic pollutants degradation	[84]
Nanobelts	800 µm × 400 nm × 20 nm	anatase	hydrothermal reaction, proton exchange, calcination	*k* = 0.0256 h^−1^ (0.000426 min) (phenol degradation in water; LED UV lamp (18 W); ca. 12 mW cm^−2^)	Photocatalysis, gas sensors, etc.	[83]
Nanorods (NRs)	~1.5 nm diameter and ~8.3 nm in length	rutile	solvothermal reaction	*k*~0.068 min^−1^ (RhB degradation, 300 W Xe lamp; full spectrum)	Organic pollutant degradation	[85]
Nanofibers/core-shell nanofibers	diameter < 100 nm	anatase, rutile, anatase + rutile	electrospinning/hydrolysis or alkoxide method	Estimated *k* = 0.027~0.1118 min^−1^ (lower values for rutile, higher values for anatase, mixed/core-shell have values in between, with values closer to the shell structure) (RhB; UV irradiation	Energy and environmental applications	[86]
Nanotubes (bamboo-type)	2.5 µm in length, 100 nm diameter	amorphous	anodization	*k* = 0.0045 min^−1^ (AO7 degradation), 0.0187 min^−1^ (MB degradation) (UV light, 200 mW HeCd laser with I = 60 mWcm^−2^, 325 nm)	Dye-sensitized solar cells, etc.	[72]
	20–60 nm diameter, 0.5–0.9 µm in length, and ~60 nm interpore distances	anatase + rutile	dynamic anodization + heat treatment	*k =* 0.0007−0.0013 min^−1^ (MB degradation, 9W UV black-light lamp (λ = 365 nm), 1 mW/cm^2^ radiation at surface)	Devices; energy and environmental applications	[87]
Nanotubes (smooth)	4.5 µm length, 45 nm diameter	anatase	anodization + heat treatment	*k =* 0.0158 min^−1^ (AO7 degradation), 0.0213 min^−1^ (MB degradation) (UV light, 200 mW HeCd laser with I = 60 mWcm^−2^, 325 nm)	Dye-sensitized solar cells, etc.	[72]
	50–60 nm diameter, 1.7–2.2 µm in length, and ~60 nm interpore distances	anatase + rutile	dynamic anodization + heat treatment	*k =* 0.0024–0.0049 min^−1^ (MB degradation, 9 W UV black-light lamp (λ = 365 nm), 1 mW/cm^2^ radiation at surface)	Devices; energy and environmental applications	[87]
Nanotubes (grown from Ti-6Al-4V)	diameter increases with anodizing potential; thickness ~280 nm	anatase (+rutile)	anodization + heat treatment	~8–42% photodegradation efficiency (depending on the anodization voltage or tube diameter); estimated *k* = 0.0005–0.003 min^−1^ (MB degradation; UV-A lamp, 0.39 W/cm^2^)	Organic pollutant degradation	[88]
Nanoribbons	200–300 nm in width; several microns in length	anatase (+rutile)	alkaline hydrothermal treatment	*k*~0.05342–0.08164 min^−1^ (RhB degradation; simulated sunlight, 300 W 230 V E27)	Organic pollutants degradation	[89]
Nanoribbons (nanopitted)	width 20~200 nm, length of 1 µm–few µm with pits of dia. 5–15 nm	TiO_2_-B	alkaline hydrothermal treatment	*k ~* 0.0024–0.011 min^−1^ (MB degradation) (natural sunlight)	Dye degradation	[90]
2D nanogrid/ nanolaces/ inverse-opal-like structure	230–610 nm diameter of holes	anatase	opal-templated sol-gel-based synthesis + heat treatment	*k*~0.022–0.058 min^−1^ (RhB degradation; 500 W Xe arc lamp, 400 nm cutoff, 25 mW cm^−2^)	Degradation of various environmental contaminants	[91]
	~150 nm diameter holes, lace thickness 10~20 nm	anatase (+rutile)	alternating-voltage anodization + heat treatment/ 2-step anodization	*k* = 0.003 min^−1^ (Cr (VI) photocatalytic reduction; simulated sunlight, 300 W xenon lamp with 100 W cm^−2^ irradiation)	Heavy metal (Cr(VI)) removal from wastewater	[92,93]
	~150 nm diameter holes, lace thickness 10~20 nm	black TiO_2_	2-step anodization process + heat-treat. in reducing atmosphere	*k* = 0.0657 min^−1^ (Cr (VI) photocatalytic reduction; simulated sunlight, 300 W xenon lamp with 100 W cm^−2^ irradiation)	Heavy metal (Cr(VI)) removal from wastewater	[93]
Nanosheets	thickness < 7 nm	anatase	hydrothermal process	*k* = 0.013 min^−1^ (RhB degradation, mercury lamp (300 W, as UV light source), 2.62 mW/cm^2^)	Renewable energy; environment	[75]
Nanoflowers	2–6 nm diameter with ~10 nm thin petals	anatase + rutile	hydrothermal + calcination	*k* = 0.03–0.12 min^−1^ (MB degradation at diff. pH; highest *k* at pH 4; UV lamp (100 W, 365 nm, 6.5 mW cm^−2^))	Effluent treatment	[94]
Dendritic nanospheres	2–3 µm diameter of entire structure; nanowire/nanoribbon spikes: 500 nm–1.5 µm long and dia. in nm	rutile	low-temperature hydrothermal method	*k*~0.018–0.024 min^−1^ (A07/RhB degradation; UVP Mineralight lamp (254 nm, 40 mW cm^−2^))	Photocatalytic membrane water purification	[73]
Nanotrees	130–180 nm nanowire diameter; 10–20 nm nanoparticle size	anatase	hydrothermal method, (1) proton exchange + calcination, (2) TiO_2_ NP decoration; sequence variation has an effect	*k* = 0.021, 0.071 min^−1^ (depending on the lattice parameter) (toluene gas decomposition; UV lamp (PL-L 18W/10/4P, Philips; 365 nm; 8.7 mW cm^−2^)	Photocatalysis, gas sensors, etc.	[95]
	Sheet-on-belt (SOB): 800 µm × 400 nm × 20 nm (trunk), up to 100 nm long (branches). Sheet-on-wire (SOW): 100 nm diameter × 800 µm (trunk) with branches up to 200 nm. Branch thickness: a few nm	mostly anatase (+a bit of rutile)	hydrothermal reaction, proton exchange, calcination, solution combustion synthesis + calcination	Up to *k* = 0.346 h^−1^ (or 0.00576 min^−1^) (SOB photocatalytic); up to *k* = 0.40 h^−1^ (or 0.0067 min^−1^) (SOW photo-electrocatalytic) (phenol degradation in water) (LED UV lamp (18 W); ca. 12 mW cm^−2^)	Environmental remediation, energy storage, green energy production	[83]
	branched nanowire; 1 µm thick, branch: 10 nm thick, 45 nm long	anatase + rutile	H_2_O_2_ oxidation, intermediate calcination, and H_2_SO_4_ treatment	*k* = 0.0007–0.0086 min^−1^ (UV + various organic compounds); 0.0057 min^−1^ (visible + RhB) (18 W UV lamp, 5 mW/cm^2^; 500 W Xe-lamp, 420 nm cut off, 200 mW/cm^2^)	Photocatalytic water-splitting, environmental remediation	[96]
Nanosheet + quantum dots (QDs)	nanosheet: thickness < 7 nm quantum dot: 3–5.6 nm	anatase	nanosheet, hydrothermal; QD, autoclave (+heating) homojunction: grinding	*k* = 0.027–0.064 min^−1^ (RhB degradation, mercury lamp (300 W, as UV light source), 2.62 mW/cm^2^)	Renewable energy technologies, environmental protection	[75]
Nanoparticles, nanobucks, and nanorods	15–25 nm NPs; 100–150 nm nanobuck; 15 nm × 150 nm nanorod	rutile + anatase	(one-step) hydrothermal synthesis	*k* = 0.033 min^−1^ (RhB degradation) (250 W Xe lamp–simulated solar light source)	Wastewater treatment	[97]
Nanorhombus nanocuboids	nanorhombus: 55~80 nm × 35~40 nm; nanocuboids: 20~30 nm × 30~50 nm	anatase	hydrothermal synthesis	*k* = 0.0134−0.0318 min^−1^ (RhB degradation; UV; simulated sunlight AM 1.5 G filter, (100 mW/cm^2^))	Dye-sensitized solar cells, etc.	[74]

^§^ acid orange 7 (AO7), rhodamine B (RhB), methyl orange (MO), methylene blue (MB), methyl red (MR).

**Table 2:** Examples of black (and colored) TiO_2_ nanomaterials and their photocatalytic performance in terms of degradation of organic compounds or hydrogen evolution.

Material	Degradation/Removal of Organics	H_2_ Generation	Reference Material/Comparison	Ref.
Black TiO_2_ nanocrystals/NPs	~7.5× faster MB degradation, solar illumination	0.1 ± 0.02 mmol h^−1^ g^−1^ (2 orders higher (solar simulator or visible IR light))	Degradation: pristine TiO_2_ nanocrystals H_2_ generation: most semiconductor photocatalysts	[194]
	Up to *k* = 0.68 min^−1^ MO degradation, 2.4× faster (simulated sunlight)	Up to 5.2 mmol h^−1^g^−1^, 1.7× faster (simulated sunlight)	Pristine P25 degradation: *k* = 0.28 min^−1^ H_2_ generation: 5.2 mmol h^−1^ g^−1^	[199]
	Up to apparent *k* (*k_app_*) = 0.998 h^−1^ or 0.0166 min^−1^ acetaminophen removal, 1.9× faster than P25 and 4.9× faster than sintered P25 (solar illum. AM 1.5G)		P25: *k* = 0.527 h^−1^ or 0.00878 min^−1^ Sintered P25: 0.203 h^−1^ or 0.00338 min^−1^	[209]
		Estimate: ~15 µmol h^−1^ g^−1^ ~5−7× higher (AM 1.5 illum.; 100 mW cm^−2^)	Anatase nanopowders; estimate: ~2−3 µmol h^−1^ g^−1^	[210]
	Anatase; ~1.5× faster, MB degradation finished in 18 min (solar illumination)		Degussa-P25: MB degradation finished in 18 min. (solar illumination)	[203]
Black hydroxylated TiO_2_ (ultrasonic.)	5.8× (solar illumination) and 7.2× (visible light) faster acid fuchsin decomposition; amorphous state		Original sol TiO_2_ (non-ultrasonically processed)	[201]
Black TiO_2_ nanotube array (TNA)	Estimate: 10~15% (3–4×) better photocatalytic degradation (brilliant blue KN-R dye; 175 W Xe lamp)		Pristine TNA	[196]
Ordered mesoporous black TiO_2_		136.2 µmol h^−1^, ~2× higher (solar)	Pristine mesoporous TiO_2_: 76 µmol h^−1^	[198]
Mesoporous black TiO_2_ hollow spheres		241 µmol h^−1^ (0.1) g^−1^, ~2× higher (solar)	Black TiO_2_ NPs: 118 µmol h^−1^ (0.1) g^−1^ Mesoporous TiO_2_ hollow spheres: 81 µmol h^−1^ (0.1) g^−1^	[197]
Defective black TiO_2_ (dimpled morphology, anodization)	High oxygen vacancy concentration (C_Vo_): up to ~80% RhB degradation (after 4 h), ~1.3× better than low C_Vo_ and ~4× better than TNA.		Low C_Vo_: up to 60% RhB degradation (after 4 h) TNA: ~20% RhB degradation (after 4 h)	[200]
Grey TiO_2_ nanoparticles (flow furnace)		Estimate: ~75 µmol h^−1^ g^−1^ ~25−37× higher (AM 1.5 illum.; 100 mW cm^−2^)	Anatase nanopowders: estimate: ~2−3 µmol h^−1^ g^−1^	[210]
Grey TiO_2_ nanoparticles (hydrogen. at high P)		Estimate: ~80−85 µmol h^−1^ g^−1^ ~27−42× higher (AM 1.5 illum.; 100 mW cm^−2^)	Anatase nanopowders: estimate: ~2−3 µmol h^−1^ g^−1^	[210]
Colored TiO_2_ (dark blue)	Up to *C/C*_0_ = 0.14 (estimated *k* = 0.197 min^−1^), 1.4× faster MO degradation (300 W, Xe lamp UV–vis light)	Up to max. prod. of 6.5 mmol h^−1^ g^−1^, 7.2× higher (UV-vis light); ~180 µmol h^−1^ g^−1^ (vis-IR)	Pristine P25 Degradation: *C/C_0_* = 0.24 (estimated *k* = 0.143 min^−1^) H_2_ generation: 0.9 mmol h^−1^ g^−1^	[195]
Blue TiO_2_(B) single-crystal nanorods	*k* = 0.0146 min^−1^; 97.01% RhB degradation (after 150 min), 6.9× and 2.1× better than TiO_2_ NPs and TiO_2_ NRs, respectively (vis light); 98.56% deg. RhB (solar light), 99.12% deg. Phenol (solar); reaction constant (*k_rxn_*) = 0.0250 (RhB) and 0.0366 (phenol), 8.8× higher than TiO_2_ NPs	Up to 149. µmol h^−1^ g^−1^ (AM 1.5 illumination), ~26.6× higher than TiO_2_ NPs	Degradation: TiO_2_ NPs: *k* = 0.0016 min^−1^; 14.06% RhB degradation (after 150 min, vis light) TiO_2_ NRs: *k* = 0.0053 min^−1^; 46.44% RhB degradation (after 150 min, vis light) H_2_ evolution: TiO_2_ NPs: 5.6 µmol h^−1^ g^−1^ (AM 1.5 illumination) TiO_2_ NRs: 40.8 µmol h^−1^ g^−1^ (AM 1.5 illumination)	[211]

**Table 3:** Steps in the mechanism for the catalytic ROS generation in the dark. Activation of H_2_O_2_ performed on TiO_2_ with single-electron-trapped oxygen vacancies (SETOVs) is proposed in ref. [221].

	Reaction *
1	H_2_O_2_ + Ti–OH → Ti–OOH + H_2_O
2	$V_{O}^{\cdot }$ + Ti–OOH → Ti–·OOH
3	$V_{O}^{\cdot }$ + Ti–·OOH → Ti–OH + ½ O_2_
4	$V_{O}^{\cdot }$ + O_2_ → ·O_2_^−^
5	$V_{O}^{\cdot }$ + H_2_O_2_ → ·OH + OH^−^
6	H_2_O_2_ + ·OH → ·OOH + H_2_O

* $V_{O}^{\cdot }$ pertains to the single electron in SETOVs.

## Data Availability

No new data were created or analyzed in this study. Data sharing is not applicable to this article.
